# *In vivo* stability of ^211^At-radiopharmaceuticals: on the impact of halogen bond formation^†^

**DOI:** 10.1039/d3md00579h

**Published:** 2023-11-23

**Authors:** Thibault Yssartier, Lu Liu, Sylvain Pardoue, Jean-Yves Le Questel, François Guérard, Gilles Montavon, Nicolas Galland

**Affiliations:** a CNRS, CEISAM UMR 6230, Nantes Université, F-44000 Nantes, France; b CNRS, SUBATECH UMR 6457, IMT Atlantique, F-44307 Nantes, France; c CNRS, IPHC UMR 7178, Université de Strasbourg, F-67037 Strasbourg, France; d Inserm UMR 1307, CNRS UMR 6075, CRCI2NA, Nantes Université, Université d’Angers, F-44000 Nantes, France

## Abstract

^211^At, when coupled to a targeting agent, is one of the most promising radionuclides for therapeutic applications. The main labelling approach consists in the formation of astatoaryl compounds, which often show a lack of *in vivo* stability. The hypothesis that halogen bond (XB) interactions with protein functional groups initiate a deastatination mechanism is investigated through radiochemical experiments and DFT modelling. Several descriptors agree on the known mechanism of iodoaryl substrates dehalogenation by iodothyronine deiodinases, supporting the higher *in vivo* dehalogenation of *N*-succinimidyl 3-[^211^At] astatobenzoate (SAB) conjugates in comparison with their iodinated counterparts. The guanidinium group in 3-[^211^At]astato-4-guanidinomethylbenzoate (SAGMB) prevents the formation of At-mediated XBs with the selenocysteine active site in iodothyronine deiodinases. The initial step of At-aryl bond dissociation is inhibited, elucidating the better *in vivo* stability of SAGMB conjugates compared with those of SAB. The impact of astatine’s ability to form XB interactions on radiopharmaceutical degradation may not be limited to the case of aryl radiolabeling.

## 1 Introduction

Only a few radionuclides are of interest for cancer treatments, and among them, ^211^At is often reported as the most potent for targeted alpha therapy (TAT) due to favorable physical properties.^1^ It decays as a 100% α-emitter with a half-life of 7.21 h, resulting in the emission of 5.9 and 7.5 MeV particles. In addition, the associated emission of X-rays (77–92 keV) offers the possibility of monitoring ^211^At distribution by SPECT imaging. It was not until the 2000s that the first two phase I trials were reported,^2,3^ proving beneficial and safe for patients. However, these trials were limited to situations in which the radiopharmaceutical is locally injected. One of the main reasons is the lack of stability of ^211^At-labeling when injection occurs intravenously, exposing the radiopharmaceutical to stronger catabolism and accelerating deastatination mechanisms.

**Chart 1:**
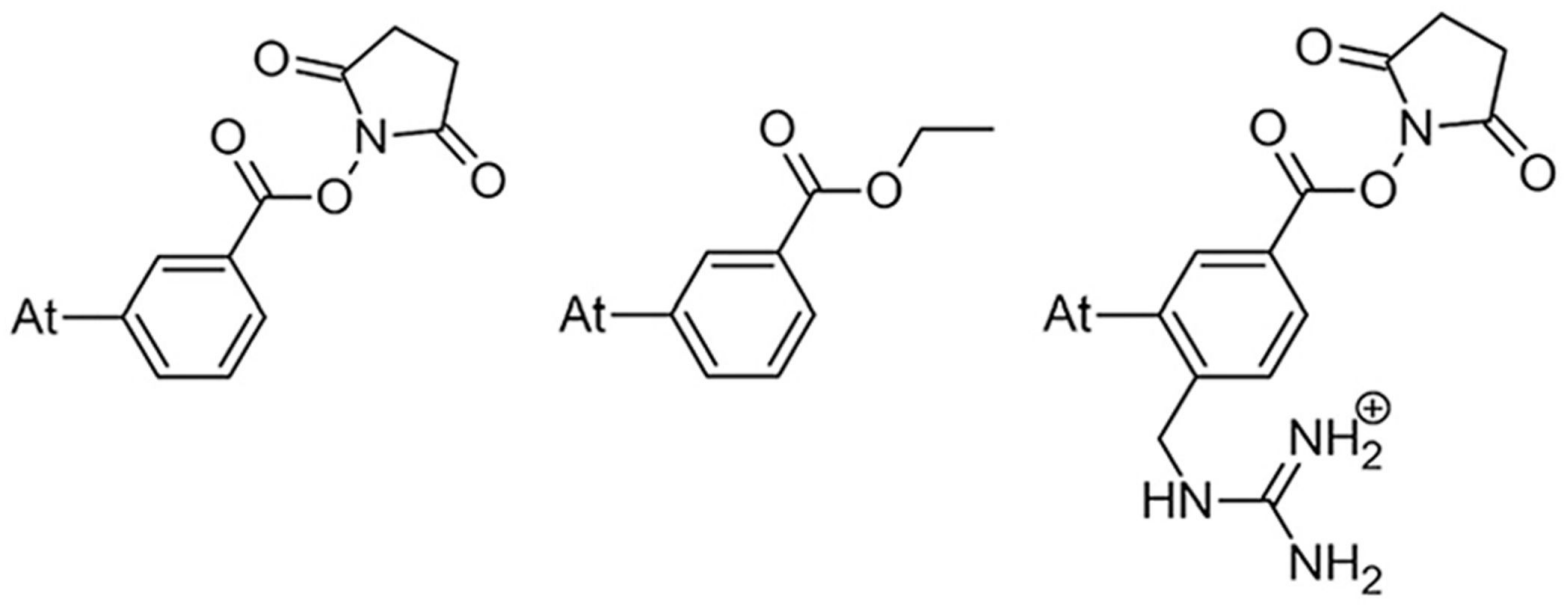
SAB (left) and SAGMB (right) astatoaryls representative of prosthetic agents, and EAB (center).

Efforts to broadly apply this radionuclide in TAT requires its coupling to a biological targeting vector, but effective labeling has long been hampered by the elusive nature of astatine.^4^ A number of its properties remain far inaccessible since it has no stable isotopes and its availability relies on artificial production by particle accelerators in transient, sub-nanomolar amounts, making usual spectroscopic tools inapplicable to chemical characterization. Consequently, the radiolabeling chemistry of ^211^At is mainly based on methods reported in the literature for radioiodination of organic compounds, iodine being astatine’s closest neighbor element. The strategy most widely used today, inspired by seminal work on the synthesis of *N*-succinimidyl 3-[^211^At]astatobenzoate (SAB, see Chart 1),^5^ involves forming a covalent bond with an aryl carbon. The resulting astatoaryl prosthetic agents can then be conjugated to the amino groups of lysine residues of carrier biomolecules. Yet, a number of preclinical studies have highlighted a significant dissociation of the At–C_Ar_ bond *in vivo*, especially with ^211^At-labeled small biomolecules or antibody fragments that are rapidly metabolized.^6,7^ Interestingly, the structure of *N*-succinimidyl 3-[^211^At]astato-4-guanidinomethylbenzoate (SAGMB, see Chart 1) is very similar to that of SAB but its *in vivo* stability is significantly higher (*i.e*. much less release of free ^211^At).^8^ The reason for the higher stability compared with SAB is however not yet rationalized.^9^

This study tackles the issue of the *in vivo* stability of astatoaryl compounds, and we propose a mechanism for the deastatination phenomenon. Understanding this mechanism at the molecular level is essential for the rational design of ^211^At-labeling procedures, and to ease the clinical transfer of radiopharmaceuticals. But until now, this subject has been little studied. At first, it was hypothesized that the strength of the covalent bond formed by astatine determines *in vivo* stability.^6,10^ The At–C bond energy increases when decreasing carbon hybridization,^11^ which supports the initial choice to favor astatoaryl compounds, but it cannot explain the difference in behavior between SAB and SAGMB conjugates. Based on the observation of accelerated ^211^At release upon carrying vector internalization within targeted cells, we have previously shown that lysosomes where are present strong oxidants could be responsible of an oxidative dehalogenation, weakening the At–C bond in astatobenzoates (*e.g*. ethyl 3-astatobenzoate, EAB in Chart 1).^12^ At is indeed much more sensitive to oxidation than other halogens,^13,14^ and the negative impact of oxidation on the *in vivo* stability of At–C_Ar_ bonds was recently confirmed.^9^ A third hypothesis is investigated in this work, where the deastatination mechanism is governed by specific interactions with protein functional groups.

**Chart 2:**
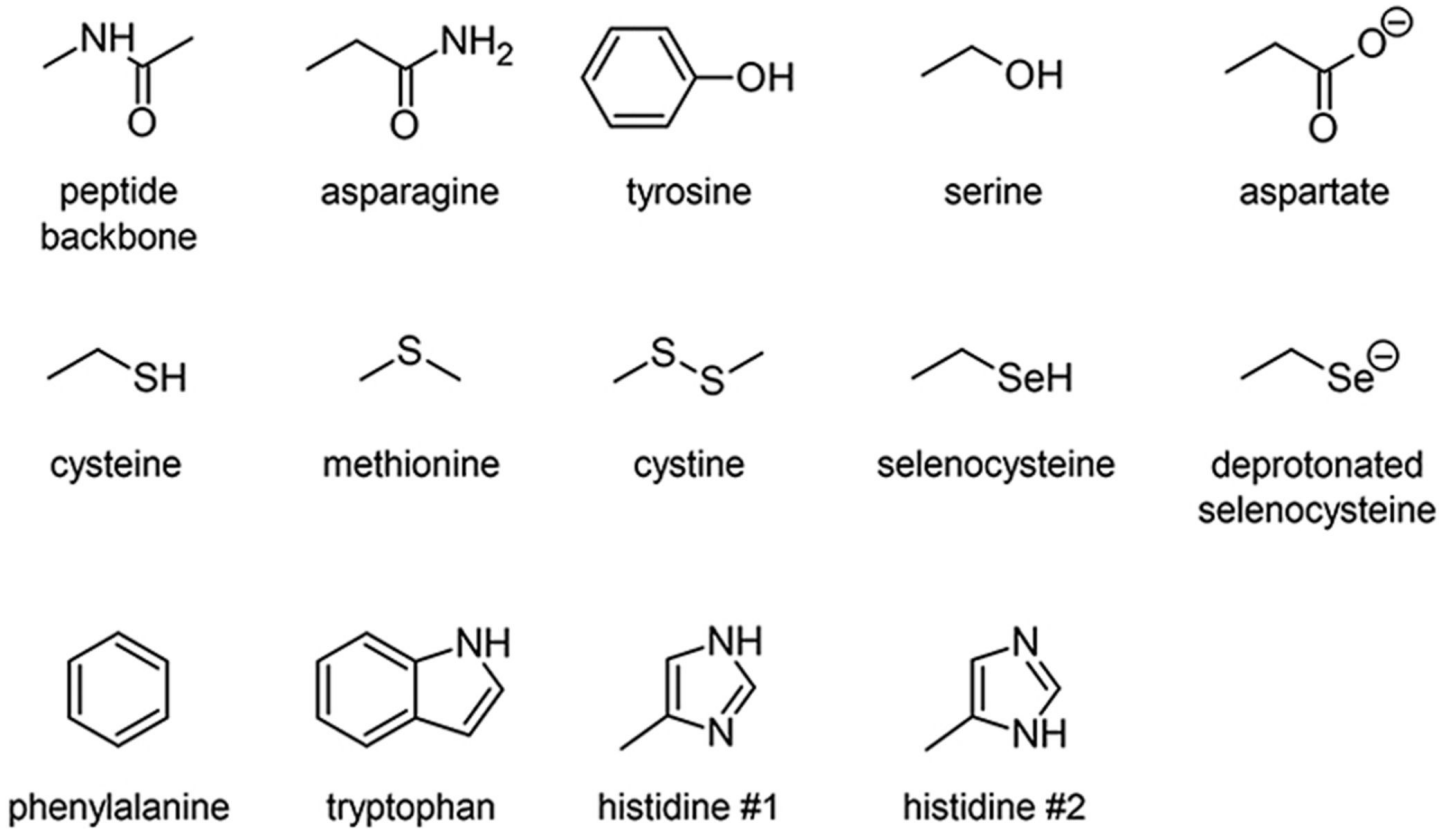
Amino acid fragments featuring the studied XB acceptor sites.

Indeed, iodoaryl substrates have long been known to undergo dehalogenation catalyzed by deiodinase enzymes.^15^ Even in the absence of the phenolic hydroxyl group required in the primary deiodination mechanisms by mammalian deiodinases, the I–C_Ar_ bond can be cleaved by type 1 and type 3 iodothyronine deiodinases.^16^ The involved mechanism is initiated by the formation of a so-called halogen-bond interaction between the iodine atom and a rare selenocysteine residue (Sec170) within the cleft of the active site.^17–19^ It is worth noting, on the one hand, that At–C bonds are weaker than their I–C counterparts,^10^ and on the other hand, that astatine is more potent for halogen bonding than iodine (see Fig. S1 in ESI† for a comparison of calculated molecular electrostatic potentials between astatobenzene and iodobenzene).^20–23^ These two arguments suggest that the mechanism introduced by Bayse and Rafferty^17^ could also apply to the *in vivo* deastatination of astatoaryl compounds. We propose to study in depth the potential of astatoaryl groups to interact *via* halogen bonds (XBs) with relevant protein functional groups, and to explain in particular the differences in stability observed in preclinical studies between SAB and SAGMB conjugates. We recently reported the first experimental characterization of XBs mediated by astatine.^24^ The technique we have since extended enables measurements in aqueous and hydrophobic media (two extremes for a biological environment),^20^ and it can be applied to characterize XBs with the nucleophilic sites representative of those of neutral amino acids. To compensate for the lack of suitable spectroscopic tools,^20,21,24^ DFT modelling has been used to obtain information on these interactions at the molecular level. The contributions of molecular modelling to the field of medicinal chemistry are well recognized,^26–28^ and, in particular, DFT calculations allow precise quantification of At-mediated XBs with anionic sites of amino acids. The chemical diversity of the selected nucleophilic sites, displayed in Chart 2, is based on a review of recurrent contacts found in PDB crystallographic structures between iodinated XB donors and protein acceptor sites.^29,30^ Chart 2 also includes the specific selenocysteine fragment of iodothyronine deiodinases involved in the above mentioned deiodination mechanism.

## 2 Results and discussion

### 2.1 Characterization of At-mediated halogen bonds with amino acids

**Fig. 1:**
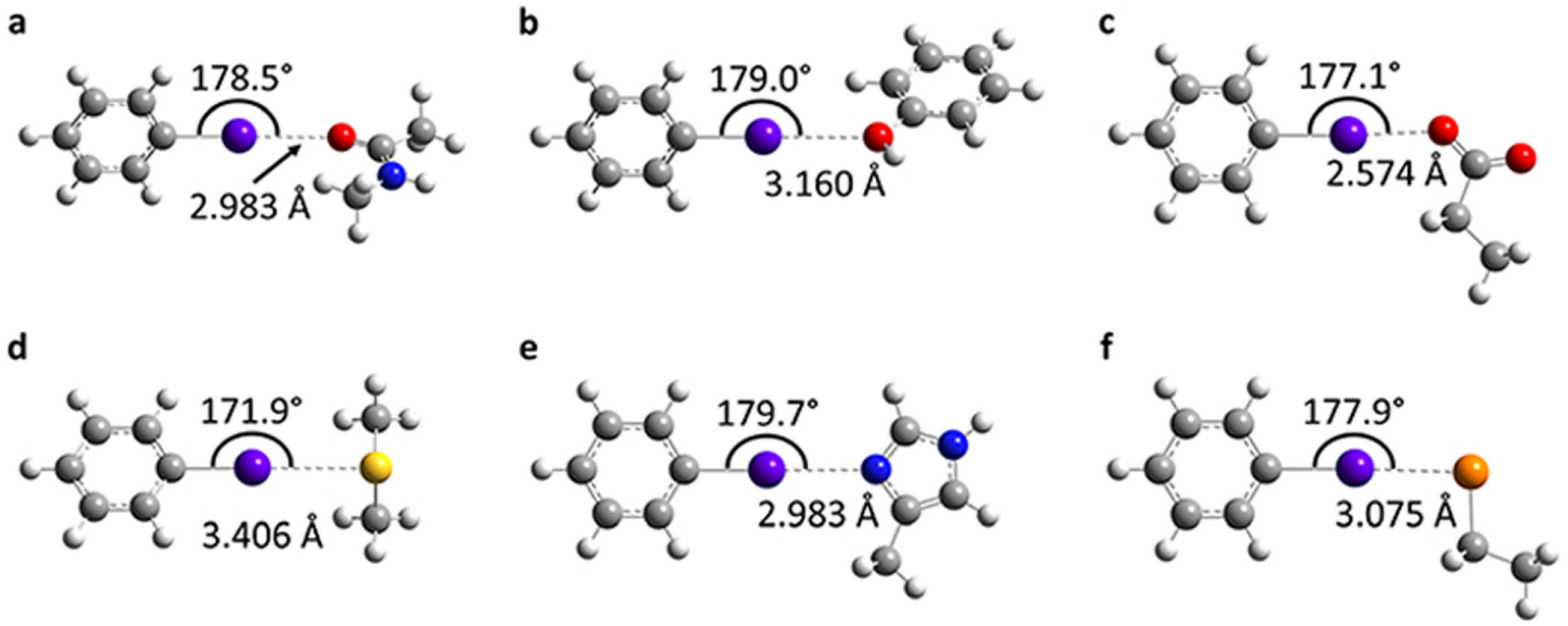
Calculated structures at the PW6B95/AVDZ level of theory for the most stable XB complexes between PhAt and nucleophilic sites of the peptide backbone (a) and of the tyrosine (b), aspartate (c), methionine (d), histidine #1 (e) and deprotonated selenocysteine (f) amino acids. Atom color codes: violet for At, red for O, blue for N, yellow for S, orange for Se, gray for C, and white for H.

**Scheme 1:**
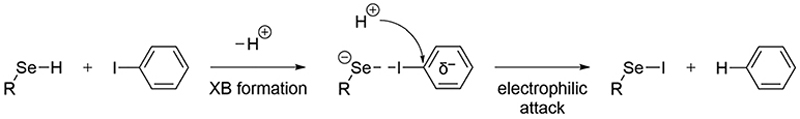
First main steps of the deiodination mechanism by type 1 and type 3 iodothyronine deiodinases.^16,25^

To determine the possible mechanisms of dehalogenation of astatoaryl compounds, it is helpful to consider at first the known mechanisms for their iodinated counterparts. Scheme 1 outlines the main steps involved in enzymatic deiodination mechanisms for iodoaryl substrates without assistance of a hydroxyl group adjacent to the halogen.^17–19,25^ It is worth noting that the selenium atom of the selenocysteine residue in iodothyronine deiodinases is deprotonated under physiological conditions.^16,31,32^ Experience shows that the mechanism for iodine removal is favorable, but is it also favorable for astatine? Let us consider the reaction shown in Scheme 1 with R = C_2_H_5_ (a simplistic carbon backbone model), for which DFT calculations helped to determine the Gibbs free energy of reaction. Substituting the iodine atom by astatine, the free energy of the reaction becomes 25.6 kJ mol^−1^ more negative (*i.e*. more favorable) at the PW6B95/TZVPD level of theory. Further calculations at the B3LYP/TZVPD level of theory confirm that the reaction is more favorable (by 25.4 kJ mol^−1^). The potential influence of an aqueous environment has also been taken into account, using the SMD solvent model. The free energy of reaction remains more favorable for At than for I, by 24.8 kJ mol^−1^. This example shows that the hypothesis of enzymatic deastatination is perfectly relevant from a thermodynamic point of view. In addition, this result corroborates the experimental observation that dehalogenation of SAB is far superior to that of its iodinated counterpart, SIB.^6,33^ We then investigated the formation of XB interactions between astatobenzene (PhAt), as an astatoaryl model, and all amino acid fragments in Chart 2. Indeed, the possibility that XB interactions with nucleophilic sites other than that of selenocysteine initiate deastatination needs to be investigated. The calculated structures of some resulting complexes are shown in Fig. 1, the others being reported in Fig. S2 (see ESI†).

The intermolecular interactions reveal geometrical features characteristic of XBs: linearity angles close to 180° (172° as a minimum), and interaction distances between 73 and 94% of the sum of the van der Waals radii of the involved atoms (see Table S1 in ESI†). The interaction energy *E*_int_, defined as the energy difference between the complex and the isolated reactants, is moderate for neutral amino acid sites. The weakest values are calculated when At interacts with N sp^2^ atoms of either the peptide backbone or the asparagine amide side chain (see Table S1†). Note that the lone pair of the nitrogen atom is conjugated with the neighboring carbonyl group, and is therefore unlikely to interact with the XB donor. Astatobenzene interacts more strongly with fragments bearing a π site, with *E*_int_ ~ −7.5 kJ mol^−1^. With nucleophilic sites consisting of S sp^3^ or O (sp^2^, sp^3^) atoms, the interaction energies almost systematically exceed −10 kJ mol^−1^. The strongest *E*_int_ value, −20.5 kJ mol^−1^, corresponds to the interaction between PhAt and the sp^2^ nitrogen of histidine.

Experimentally, we have also explored the ability of astatoaryl compounds to interact by XB with neutral nucleophilic amino acid sites. Ethyl 3-astatobenzoate (EAB), which only differs from SAB in the substitution of the coupling function (succinimide), was selected as XB donor. The aim is to determine complexation constants using a liquid/liquid competition method in which we have extensive experience.^4,24,34,35^ In particular, this method has enabled us to establish the first halogen-bond basicity scale that is specific to astatine, p*K*_BAtI_.^20^ It combines the equilibrium constants measured for the complexation between astatine monoiodide (AtI) and sixteen neutral Lewis bases. The p*K*_BAtI_ scale culminates with *N*,*N*,*N*′,*N*′-tetramethylthiourea (TMTU) as the strongest XB acceptor, particularly in comparison with the nucleophilic sites of selected neutral amino acids (Chart 2). The method’s principle is to track ^211^At distribution ratio (*D*) between an aqueous phase and an organic phase (cyclohexane solvent) as a function of the introduced concentration of the Lewis base:

$$D=\frac{A_{\text{org}}V_{\text{aq}}}{A_{\text{aq}}V_{\text{org}}}$$

where *A* values correspond to the activities measured in organic and aqueous phases, and *V* values correspond to the volume of the organic and aqueous phases. If a change in *D* occurs, there must be a change in astatine speciation in the system, *i.e*. EAB reacts with the studied Lewis base. This change can be reproduced quantitatively from thermodynamic models so as to derive the value of the complexation constant *K*.

Taking advantage of our previous practice in the synthesis and radiochemical studies of EAB,^12^ we first investigated its interaction with the strongest Lewis bases. In particular, TMTU has the potential to form At-mediated complexes in both organic and aqueous phases (apolar and polar surroundings).^20^ However, when EAB and TMTU are brought into contact, the distribution ratio *D* remains constant while the TMTU concentration is increased, as shown in Fig. S3 (see ESI†). The unchanged *D* as a function of TMTU concentrations reflects either no formation of XB complexes involving EAB, or the interaction is too weak to arise a visible change in *D* within the investigated experimental conditions. In the latter case, it is not possible to determine a formation constant, but we can give an estimate of its upper limit. As discussed in ESI,† the modelling of behavior of *D* suggests a *K*_EAB_ in cyclohexane smaller than 10^1.0^ and a *K*_EAB_ in water smaller than 10^0.5^. We have checked this result by means of DFT calculations, which have shown that *K* values of At-mediated XBs can be predicted with greater accuracy than the average experimental uncertainty (0.47 in logarithm unit).^20^ Let us consider the following exchange reaction of XB donors:

$$\text{TMTU}\cdots \text{AtI}+\text{EAB}\overset{\text{Δ}G(1)\;}{\to }\text{TMTU}\cdots \text{EAB}+\text{AtI}$$

From the calculation of the Gibbs free energy of this reaction, and knowing the precise value of the complexation constant between AtI and TMTU, we can deduce the complexation constant between EAB and TMTU: *K*_EAB_ = exp(−Δ*G*(1)/*RT*)·*K*_AtI_. Based on PW6B95/TZVPD calculations and a value of 10^5.69±0.32^ measured for *K*_AtI_ in cyclohexane,^20^ it comes *K*_EAB_ = 10^0.01±0.32^. The situation is even less favorable in an aqueous environment. The *K*_AtI_ value measured in water is 10^4.40±0.09^,^20^ leading for *K*_EAB_ to a value of 10^−4.92±0.09^. Experiments and calculations agree that the halogen bond between EAB and TMTU, with a calculated interaction energy about −22 kJ mol^−1^, is not strong enough to sufficiently stabilize the complex.

Note for the XB between PhAt and TMTU, the interaction energy is very close, about −20 kJ mol^−1^. It confirms that astatobenzene is a relevant model of SAB derivatives in terms of their ability to form XBs. Since the XBs between PhAt and neutral amino acid sites are all calculated to be of equal or lesser strength (*cf*. Table S1†), it seems unlikely that this type of interaction could control alone the binding of astatoaryl compounds to enzymatic receptor sites. Conversely, this is much more plausible when it comes to interactions with anionic residues. Strong values of *E*_int_ have been calculated for the XBs between PhAt and the fragments of aspartate and deprotonated selenocysteine, −77.9 and −86.6 kJ mol^−1^ (Table S1†), respectively.

### 2.2 Implications for the dehalogenation mechanism

**Fig. 2:**
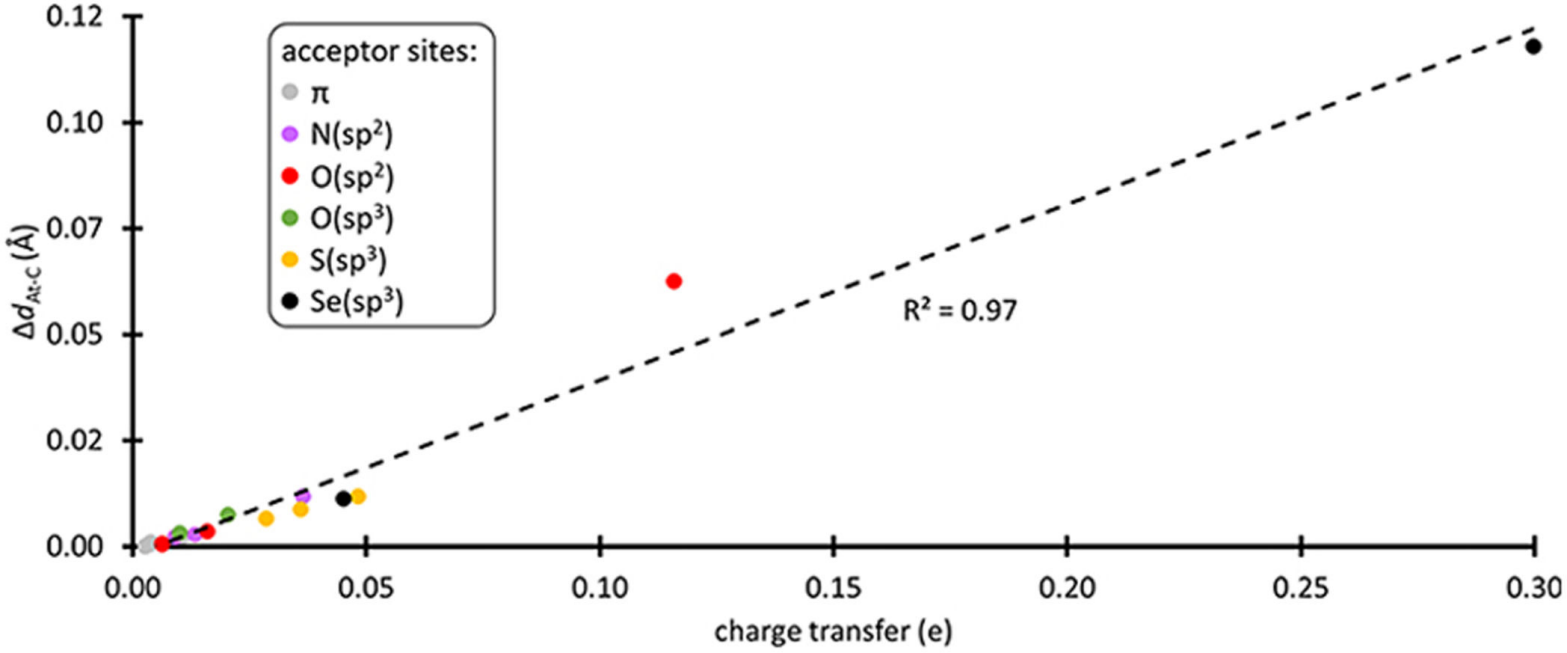
Correlation between the At–C bond lengthening and the charge transfer in the most stable XB complexes between PhAt and amino acid fragments (data sorted according to the type of acceptor site).

XB interactions are known to be mainly electrostatic in nature, but the contribution of charge transfer from the acceptor to the XB donor is not negligible,^36^ particularly for the most polarizable halogens such as astatine.^37,38^ In the complexes formed with the amino acid fragments, the electronic distribution at PhAt was quantified using the natural population analysis (NPA) method.^39^ In ESI,† Fig. S4 shows the evolution of the amount of electron transferred to PhAt, which can reach 0.30 e with deprotonated selenocysteine as XB acceptor. The general trend is an increase in interaction energy with charge transfer (82% of the variation of *E*_int_ is explained by the charge transfer at the PW6B95/TZVPD level of theory). According to the concept of halogen bonding, electron density is transferred to the anti-bonding At–C molecular orbital, which should lead to a weakening of the At–C bond. As shown in Fig. 2, there is a strong linear relationship between the lengthening of this bond (Δ*d*_At–C_) in the XB complexes and the charge transfer (coefficient of determination of 0.97). Δ*d*_At–C_ is less than 0.5% when PhAt interacts with neutral amino acid sites, but is much greater for anionic sites. The At–C bond lengthening is 0.113 Å for the case of deprotonated selenocysteine. In line with the enzymatic dehalogenation mechanism proposed by Bayse and Rafferty,^17^ the XB formation occurring during the first step (*cf*. Scheme 1) does not directly break the carbon–halogen bond. However, the bond appears notably weakened in case of a XB involving an anionic residue. In addition, a negative charge is effectively transferred to the aryl, which prepares the electrophilic attack in the second step.

The attack of a proton donor on the carbon bearing the halogen atom, the *ipso* carbon (C_*ipso*_), then leads to the breaking of the carbon–halogen bond, simultaneously with the formation of a carbon–hydrogen bond.^16,17^ This rate-determining step is controlled by the potential of C_*ipso*_ for an attack by a proton donor. It is therefore essential to evaluate the C_*ipso*_ activation by the XB formed during the first step of the mechanism, *i.e*. quantifying the nucleophilic character of this carbon in previous XB complexes. Atomic partial charges are still a widely used descriptor of reactivity in chemistry. There is a good consistency in the calculated values for the ring carbon atoms between PhAt and EAB, *i.e*. the experimental model of SAB (see Fig. S5 in ESI†).

**Fig. 3:**
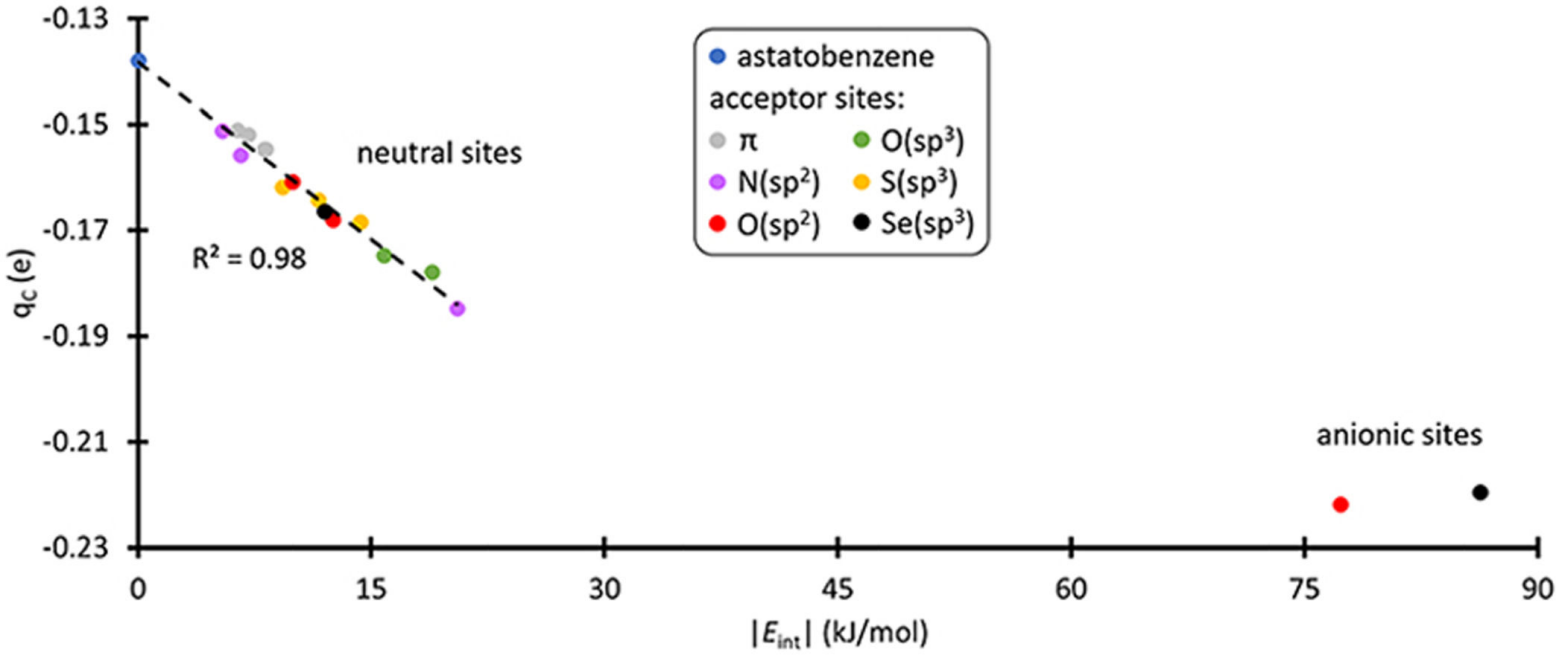
Partial charge of C_*ipso*_ and interaction energy calculated at the PW6B95/TZVPD level of theory, in the most stable XB complexes between PhAt and amino acid fragments (the data are sorted according to the type of acceptor site).

C_*ipso*_ appears as the least nucleophilic one with a partial charge of −0.14 e at the PW6B95/TZVPD level of theory. Fig. 3 shows the evolution of the atomic charge of C_*ipso*_ (*q*_C_) in the XB complexes. *q*_C_ becomes more negative as the strength of the XB interaction increases, indicating an enhancement of C_*ipso*_ nucleophilic character. This carbon is indeed activated. More precisely, when PhAt interacts with neutral amino acid sites, the *ipso* carbon charge evolves proportionally with the interaction energy. For the anionic sites, a different behavior appears with *q*_C_ that saturates at −0.22 e. This corresponds to a 57% increase in charge compared with the isolated astatobenzene, demonstrating that the two anionic amino acids unambiguously lead to the most pronounced nucleophilic character.

**Fig. 4:**
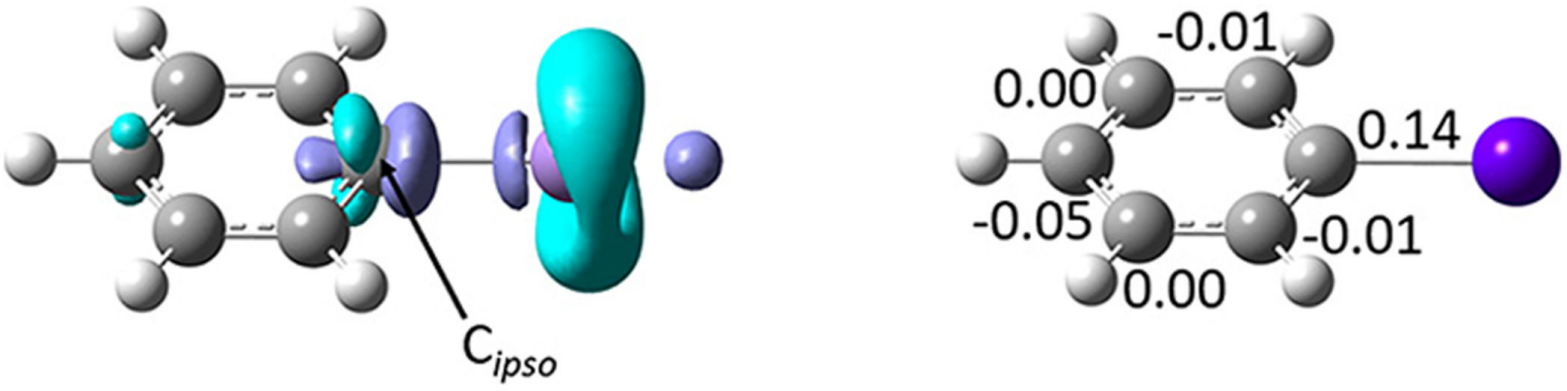
Left: Calculated isosurfaces of the dual descriptor for astatobenzene (purple for Δ*f* = 0.0045 a.u., cyan for Δ*f* = −0.0045 a.u.), and right: condensed-to-atom values of the dual descriptor (in a.u.) for the ring carbon atoms.

A more complete picture of astatobenzene reactivity is provided by the dual descriptor, Δ*f*.^40^ The dual descriptor is rooted in conceptual DFT,^41^ which focuses on extracting information in terms of local reactivity by the calculation of relevant quantities designed from first principles, often responses of the molecular compound to a (chemical) perturbation. Δ*f* expresses the local response of the Fukui function upon a variation of the number of electrons. Predominant electrophilic and nucleophilic local tendencies are thus characterized at once according to the sign and magnitude of the dual descriptor. Fig. 4 shows the regioselectivity for PhAt of the nucleophilic (Δ*f* < 0) and electrophilic (Δ*f* > 0) sites. C_*ipso*_ features the strongest nucleophilic site (cyan-colored lobes with π symmetry) of the ring carbons, but also the most electrophilic sites (purple-colored lobes). The condensed-to-atom values of the dual descriptor, presented in Fig. 4, indicate that C_*ipso*_ is predominantly electrophilic (Δ*f*_C_ = 0.14 a.u.) while the other carbons appear rather nucleophilic. When PhAt interacts with neutral amino acid fragments, Δ*f*_C_ decreases only slightly (see Fig. S6 in ESI†), demonstrating low C_*ipso*_ activation. The possibility that a deastatination mechanism is initiated by the formation of an XB with a neutral enzymatic site can definitely be ruled out. Despite a significant decrease in Δ*f*_C_ (Fig. S6†), its value of 0.06 a.u. would indicate that the electrophilic character of C_*ipso*_ persists to dominate when the astatobenzene interacts with the negatively charged oxygens of aspartate. Only the XB complex with the deprotonated selenocysteine site reveals an *ipso* carbon that has become predominantly nucleophilic, with Δ*f*_C_ = −0.11 a.u. The selenocysteine residue would almost certainly lead to the most important activation of C_*ipso*_, and help for the second step of the dehalogenation mechanism.

**Scheme 2:**
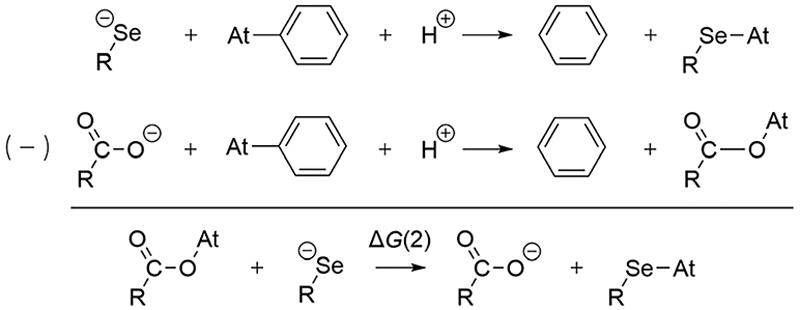
Competition between deastatination reactions by selenocysteine and aspartate residues.

The preference for a selenocysteine residue over an aspartate residue has been confirmed from a thermodynamic point of view. Let us consider the deastatination reactions involving these two residues, following the mechanism in Scheme 1. As shown in Scheme 2, comparing the respective chemical equations is equivalent in studying a halogen exchange reaction whose standard free energy of reaction is Δ*G*(2). With R = C_2_H_5_ (a simplistic carbon backbone model), the PW6B95/TZVPD calculations yield a deastatination reaction by selenocysteine that is 122.8 kJ mol^−1^ more favorable than the one by aspartate. The potential influence of an aqueous environment was introduced by means of the SMD solvent model. Δ*G*(2) is then −143.3 kJ mol^−1^, *i.e*. an even stronger preference for deprotonated selenocysteine. In summary, the dehalogenation reaction of an astatoaryl group has been shown to be thermodynamically more favorable than that of its iodinated counterpart, and key features of the original mechanism introduced by Bayse and Rafferty^17^ for iodoaryl compounds have been identified in the case of deastatination. The formation of an XB interaction weakens the At–C_Ar_ bond without breaking it, but more importantly activates the *ipso* carbon for subsequent electrophilic attack to release the halogen atom. All the descriptors of the enzymatic mechanism are maximal when the interaction involves a selenocysteine residue, *i.e*. the deprotonated selenium site already described for the deiodination reaction. It is unlikely that XBs with other amino acid sites could induce deastatination of astatoaryl compounds.

### 2.3 On the *in vivo* stability of SAGMB conjugates

**Chart 3:**
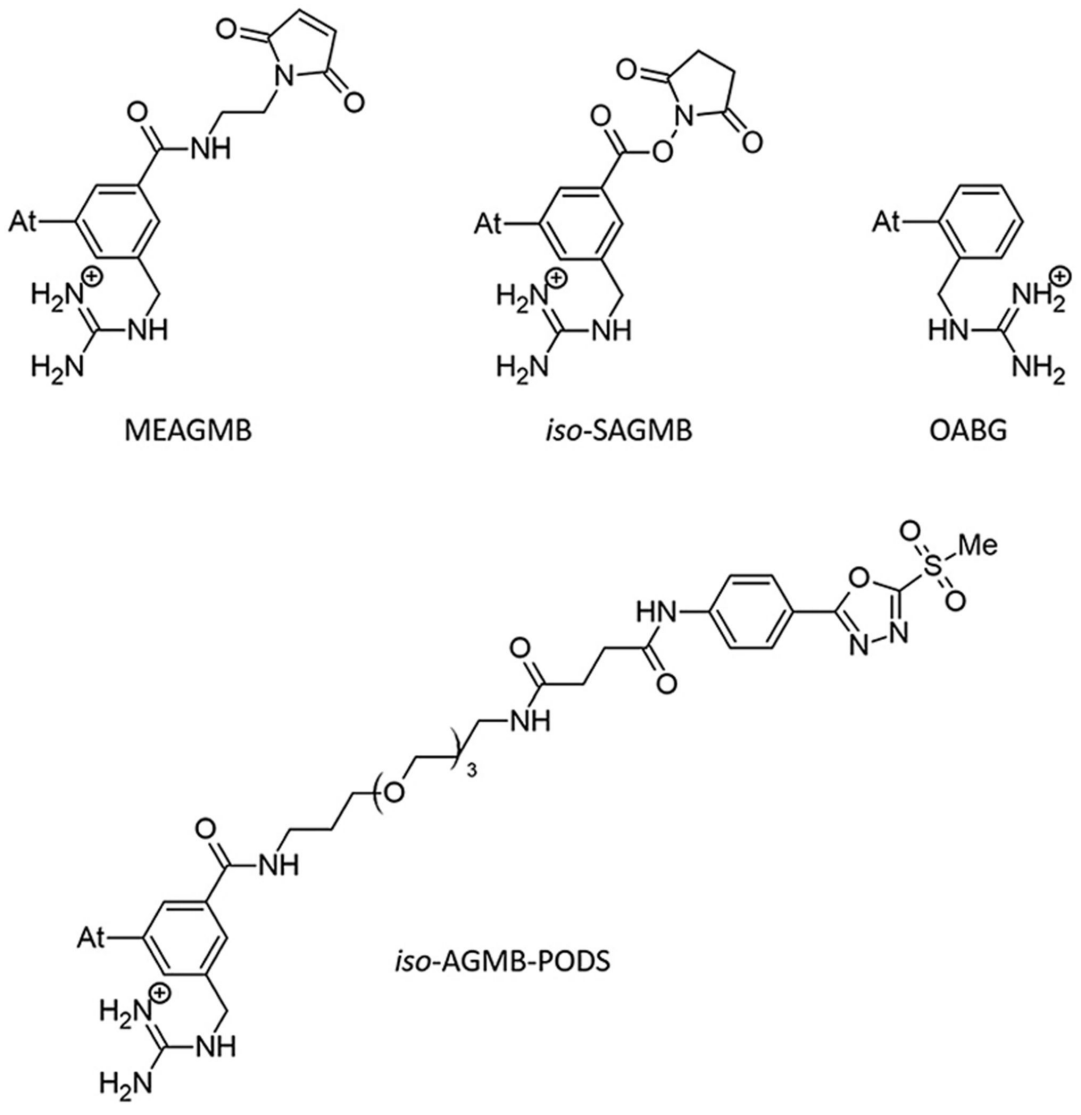
SAGMB-derived prosthetic agents and model used (OABG).

The mechanism of dehalogenation of astatoaryl groups initiated by a XB interaction with specific iodothyronine deiodinases, presented herein, can explain that *in vivo* dehalogenation of SAB is far superior to that of its iodinated counterpart, SIB.^6,33^ At first glance, however, this mechanism seems unable to rationalize the difference in stability between compounds such as SAB and SAGMB conjugates. 20 years after publication of the synthesis of SAGMB (Chart 1),^42^ which has recently led to innovative derivatives as promising prosthetic agents (iso-SAGMB, MEAGMB, iso-AGMB-PODS),^8,43,44^ its relatively good *in vivo* stability remains unexplained.^9^ As shown in Chart 3, iso-SAGMB, MEAGMB, and iso-AGMB-PODS all carry a guanidinium group in the vicinity of the astatine atom. In line with the direct comparison of *in vivo* stabilities between SAGMB and SAB,^8^ the coupling function of SAGMB was removed as for SAB in order to obtain a model compound that could be compared with PhAt. The resulting compound is 1-(*o*-astatobenzyl)guanidine (OABG, see Chart 3).

**Fig. 5:**
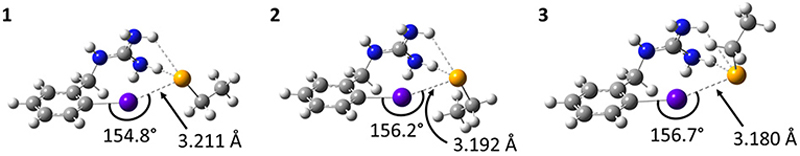
Calculated structures at the PW6B95/AVDZ level of theory for the XB complexes between OABG and the deprotonated selenocysteine fragment. Atom color codes: violet for At, blue for N, orange for Se, gray for C, and white for H.

Note that for OABG, the bond dissociation enthalpy of At–C is around 198 kJ mol^−1^, which is very close to that calculated for PhAt (195.4 kJ mol^−1^). The presence of the guanidinium group has little impact on the At–C bond strength, and the latter is therefore unlikely to explain the significant difference in stability between SAGMB and SAB. Let’s investigate how the selenocysteine-specific reaction mechanism might rationalize the impact of the guanidinium group on deastatination. It is worth remembering that this mechanism is initiated by the formation of an XB interaction between the halogen and a deprotonated selenium atom. The XB complexes formed between OABG and the selenocysteine fragment are shown in Fig. 5. The XB interaction distances are about 0.12 Å longer than in the analogous complexes formed by PhAt. The weighted value of the total interaction energy, according to the Boltzmann populations of the three complexes, is however −423.5 kJ mol^−1^. This far exceeds the previously calculated value of −86.6 kJ mol^−1^ for the complexes between PhAt and the same selenocysteine fragment. The difference (~337 kJ mol^−1^) can be explained by the existence, beyond the XB interaction, of a strong electrostatic interaction between the positive guanidinium group and the negatively charged selenium atom. This charge–charge interaction largely dominates the energetic contribution of halogen bonding, and appears mediated by hydrogen bonds (HBs) involving amine group hydrogens (Fig. 5).

**Fig. 6:**
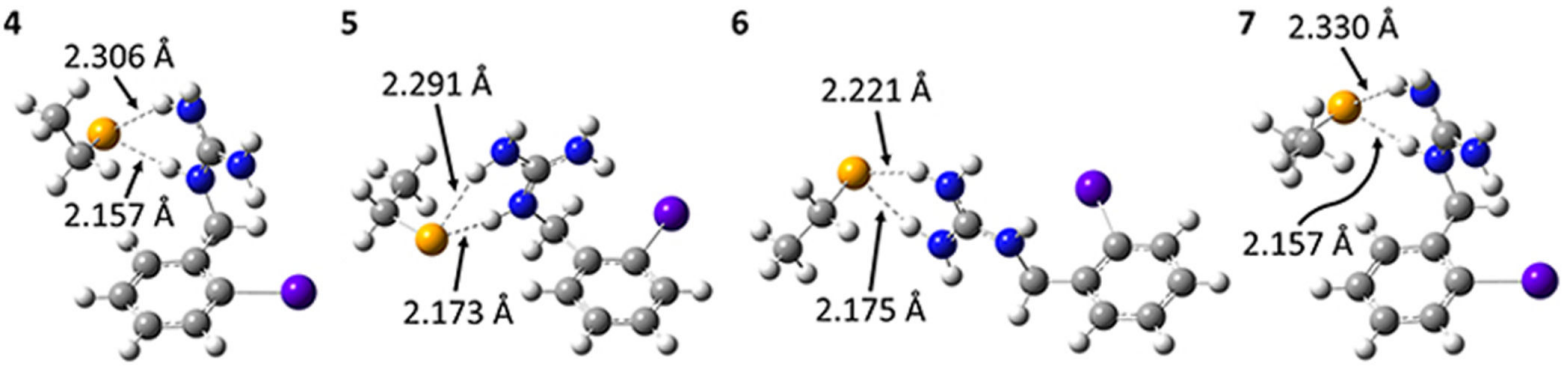
Calculated structures at the PW6B95/AVDZ level of theory for the most stable HB complexes between OABG and the deprotonated selenocysteine fragment. Atom color codes: violet for At, blue for N, orange for Se, gray for C, and white for H.

Other complexes stabilized solely by the charge–charge interaction were also found, the most stables ones being shown in Fig. 6. The interaction is again mediated by HBs, since the positive charge is delocalized over the entire guanidinium group. Hence, the negatively charged selenium atom interacts with different hydrogens of the guanidinium group. According to the HB interaction distances, ranging from 72 to 78% of the sum of the van der Waals radii of the involved atoms, these interactions are strong. They are so strong (weighted interaction energy of −427.7 kJ mol^−1^) that complexes **4**–7 would be the only ones present among all possible stable structures between OABG and the selenocysteine site. Table 1 presents the calculated relative stabilities for all presented complexes at the PW6B95/TZVPD level of theory, together with the corresponding Boltzmann populations. **4, 5, 6** and **7** respectively account for 40, 32, 24 and 4% of all complexes. The effective probability of the formation of XB complexes would be nearly zero, and consequently the enzymatic deastatination mechanism could not be triggered.

This possibility was also investigated for hydrophilic and polar conditions. For each complex, its Gibbs energy of solvation in aqueous phase was calculated. The results for the main complexes are shown in Table 1, with Boltzmann populations recalculated for these conditions. It is confirmed that the population of XB complexes is nearly zero, the only present complexes in aqueous medium being stabilized solely by the charge–charge interaction. Irrespective of the environment, no XB interaction would therefore be expected between the OABG motif and the deprotonated selenocysteine site. The introduction of the guanidinium group at the aromatic ring leads to a preferred charge–charge interaction (between amine group hydrogens and the negatively charged selenium atom). This prevents the formation of XBs, and thus the initiation of the dehalogenation mechanism introduced by Bayse and Rafferty.^17^ This role attributed to the guanidinium group can explain the greater *in vivo* stability of SAGMB compared with SAB,^8^ and more generally the good stability of SAGMB derivatives.^8,43,44^ In addition, the further improved *in vivo* stability of iso-SAGMB over SAGMB,^43^ resulting from placing the guanidinium group in the *meta* position relative to astatine, may have a straightforward explanation. When the guanidinium group forms the charge–charge interaction, it moves the astatine further away from the active site of the selenocysteine residue such that structures like those shown in Fig. 5 (simultaneous XB and charge–charge interactions) are more unlikely because of greater geometrical constraints. The potential for XB interaction with iodothyronine deiodinases is as a consequence further reduced.

## 3 Conclusion

Today, many radiopharmaceutical candidates whose radiolabeling is based on At–C_Ar_ bond formation exhibit suboptimal *in vivo* stability, which leads to detrimental release of free ^211^At. The hypothesis that the formation of halogen bonds (XBs) with protein functional groups initiates a dehalogenation mechanism, as previously proposed and experimentally demonstrated for iodoaryl substrates,^17,18^ was investigated. We have finely characterized the influence on astatoaryl models of forming XB interactions with the nucleophilic sites of 12 representative amino acids. Several descriptors corroborate the enzymatic mechanism of deiodination, such as the lengthening of the At–C_Ar_ bond, indicating a weakening of the latter, and the activation of the *ipso* carbon by strengthening its nucleophilic character. Experimental and DFT modelling results suggest that At-mediated XBs are surely weak with neutral amino acid sites and irrelevant. A thermodynamic analysis based on the free energies of reaction involving the anionic sites of aspartate and selenocysteine, tends to minimize the importance of the aspartate residue. In the end, deprotonated selenocysteine is the residue suspected of causing deastatination, in line with the dehalogenation mechanism of iodoaryl substrates by iodothyronine deiodinases.

Following the presented deastatination mechanism, the astatine’s ability to form stronger XBs and the more favorable calculated reaction energy support the *in vivo* dehalogenation of *N*-succinimidyl 3-[^211^At]astatobenzoate (SAB) conjugates far superior to that of their iodinated counterparts (SIB conjugates).^6,33^ Furthermore, regarding the difference in *in vivo* stability between SAB and *N*-succinimidyl 3-[^211^At] astato-4-guanidinomethylbenzoate (SAGMB) derivatives, still unexplained to date, a connection is revealed with the astatine’s ability to form XBs. The SAB and SAGMB structures differ in the introduction of a guanidinium group at the aromatic ring, which leads to a preferred charge–charge interaction with the negatively charged selenium atom of the selenocysteine residue. This prevents the formation of a XB, and thus the deastatination mechanism of the astatoaryl group in SAGMB cannot begin. XB interactions between astatine and protein receptors have therefore a major impact: their formation or absence would condition the *in vivo* stability of radiopharmaceutical candidates when astatine is attached to an aryl carbon.

**Chart 4:**
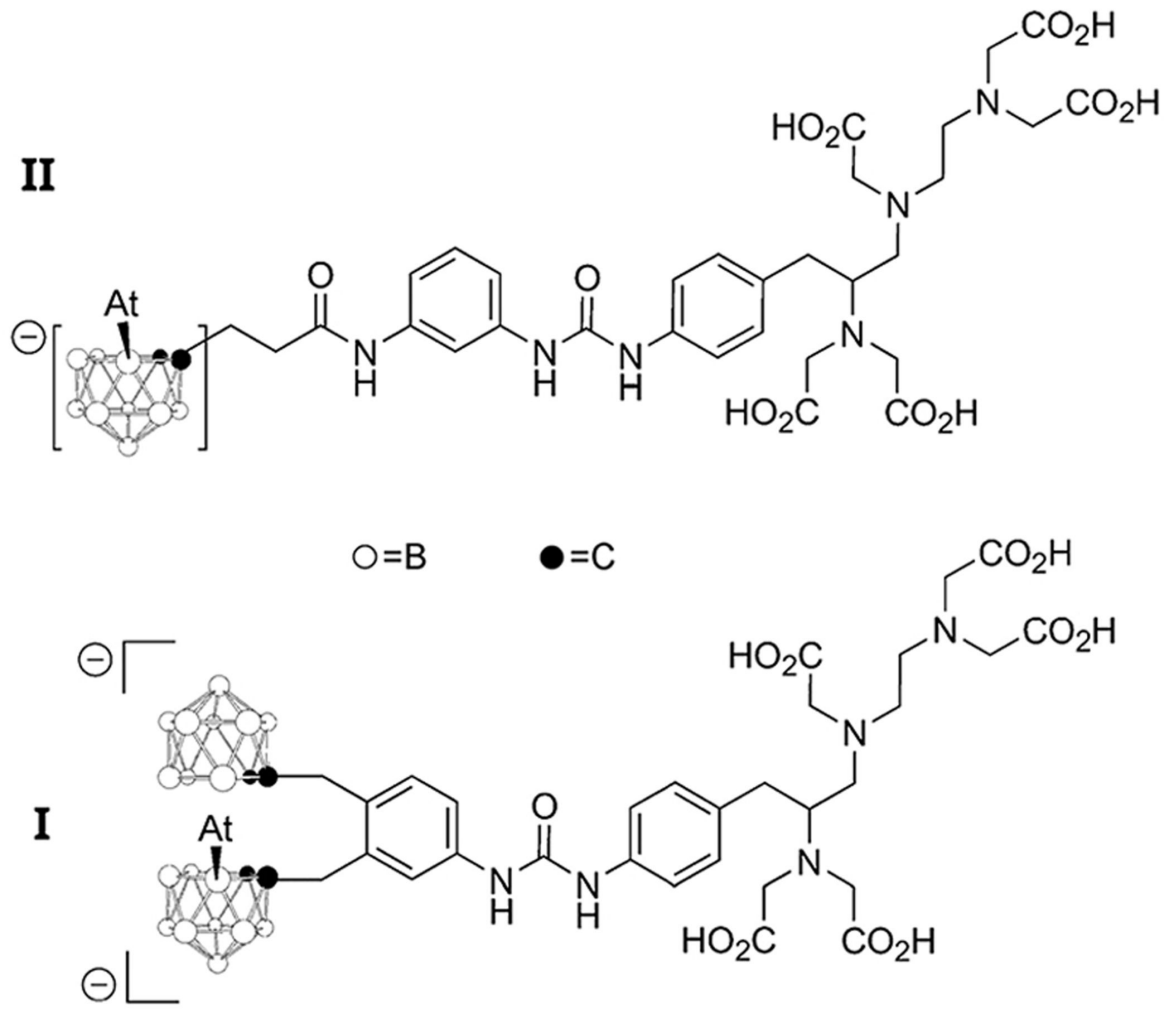
Two ^211^At-labeled boron clusters evaluated for TAT.

This issue is probably also important in other types of radiolabeling with ^211^At. The most successful alternative to date relies on the formation of a covalent At–B bond with boron clusters.^4,6^ In the quest to design efficient reagents for attaching ^211^At to carrier biomolecules, the two astatinated *nido*-carborane derivatives displayed on Chart 4 deserve a special attention. It was notably demonstrated that the bis-*nido*-carboranyl derivative **I** exhibits a much higher stability towards *in vivo* deastatination than the mono-*nido*-carboranyl derivative **II**.^45^ Although it remains to be rationalized, this difference could originate from a stabilizing XB interaction in **I** between ^211^At and the second negatively charged *nido*-carborane group (acting as a Lewis base) to bridge both *nido*-carborane moieties. Once again, it seems that controlling the astatine’s ability to interact by XB protects the At–B bond from *in vivo* dissociation. Such hypothesis is currently under active investigations.

## 4 Materials and methods

### 4.1 Experimental procedures

#### Production of ^211^At

^211^At was produced through the ^209^Bi(α, 2n)^211^At nuclear reaction at the ARRONAX cyclotron (Nantes, France). ^209^Bi targets were irradiated by alpha external beams accelerated and downgraded to 28.6 MeV. After irradiation, astatine was extracted by a dry distillation method, and, finally, ^211^At was recovered in chloroform with a specific activity close to 500 MBq mL^−1^. The radiological purity of ^211^At was verified by γ-ray spectroscopy with a high purity germanium (HPGe) detector.

#### Synthesis and purification of EAB

Two different approaches were used and the EAB purity was checked by HPLC after every step for both synthesis approaches.

##### Method 1

The synthesis of EAB was done following the previously described methods in ref. 12. Briefly, 10 μL of acetic acid, 25 μL of 2 mg mL^−1^ of *N*-chlorosuccinimide in methanol, and 1.1 mg of ethyl 3-(tri-*n*-butylstannyl) benzoate in 25 μL of methanol were mixed in an HPLC vial. Then 50 μL of ^211^At in chloroform were added (roughly corresponding to 5–10 MBq of activity). After 20 min incubation at ambient temperature, EAB was purified by HPLC using a Dionex Ultimate3000 HPLC device with an Interchrom C18 column piloted by the Chromeleon 6.80 software (ThermoFisher Scientific Inc.). It was coupled with a gamma detection system (Raytest GABI Star) piloted by the Gina software (Raytest Isotopenmeßgeräte GmbH). EAB fraction (~150 μL) was recovered together with acetonitrile/methanol eluent, and the eluent was then evaporated by a nitrogen gas flux. Finally, EAB was recovered in Milli-Q water, ready for the study of reactivity with TMTU.

##### Method 2

The synthesis of EAB was done following the iodonium salt method.^46^ Briefly, the 100 μL of chloroform containing about 10 MBq of ^211^At was evaporated. Then, the dry astatine is solubilised in 100 μL of 10^−2^ mol L^−1^ sodium acetate at in water. 900 μL of the iodonium salt precursor is added and the mixture is stirred at 60 °C to form EAB. The EAB is purified by Sep-Pak® Cartridges and collected in 500 μL of acetonitrile. The solvent is evaporated by a nitrogen gas flux and the dry EAB is recovered using 500 μL of cyclohexane for the study of reactivity with TMTU.

#### Liquid/liquid competition method

The liquid–liquid extraction protocol used for studying the reaction has been described previously in ref. 20 and 21. Two liquid immiscible phases were prepared: the organic phase was cyclohexane containing TMTU, and the aqueous phase was Milli-Q water. EAB was either doped in cyclohexane or water according to its synthesis method. After preparation, 2 mL of the organic phase and 4 mL of the aqueous phase were put together in a Pyrex tube (with activity about 1000 Bq), and were shaken for 2 h to reach the reaction equilibrium. The two phases were then separated and 1 mL of aliquot of each phase was taken to measure their radioactivity by a liquid scintillation counter. For a set of experiment, only the initial concentration of TMTU varied (from 3 × 10^−7^ to 10^−2^ mol L^−1^). The experiment was repeated twice with different ^211^At sources.

### 4.2 Molecular modelling

The two-component (2c) relativistic density functional theory (DFT) was proven to be accurate for investigating At-containing species.^47,48^ The generalized Kohn–Sham (GKS) method of the Gaussian 16 program^49^ takes advantages of relativistic pseudo-potentials containing scalar and spin-dependent terms to treat the electron correlation and the relativistic effects on an equal footing. We opted for the global hybrid global hybrid B3LYP and PW6B95 functionals, which are two of the very best among 36 DFT methods recently tested in a benchmark study focusing on At-species.^48^ The small core pseudo-potentials ECP*n*MDF with *n* = 10, 28 and 60 were used for the Se, I and At atoms, respectively.^50,51^ Two sets of basis functions were used for describing the explicitly treated electrons. The AVDZ set of double zeta quality, selected solely for the geometry optimizations and subsequent frequency calculations, combines the aug-cc-pVDZ-PP basis sets^50,51^ supplemented with two-component extensions for the At (ref. 47) and I (ref. 52) atoms, the aug-cc-pVDZ-PP basis set for Se,^50^ and the cc-pVDZ basis sets for the remaining atoms, augmented with diffuse functions for non-H atoms.^53–55^ The TZVPD set of triple zeta quality, used solely for single-point energy calculations on previously optimized geometries, combines the dhf-TZVPD-2c basis sets for the At and I atoms,^56^ and the def2-TZVP basis sets for the remaining atoms,^57^ augmented with diffuse functions for non-H atoms.^58^ Energies of the halogen bonded complexes were corrected from the basis set superposition error using the counterpoise method.^59^

For each species, its Gibbs free energy at *T* = 298.15 K is estimated using (i) the energy computed with one DFT functional (B3LYP or PW6B95) and the TZVPD basis set, and (ii) the thermodynamic corrections from the frequency calculation performed with the same functional and the AVDZ basis set. Note that some species exhibit several competitive conformers, their Gibbs free energies have been then evaluated using a Boltzmann distribution:

$$G_{\left\{x\right\}}=-RT\;\text{ln}\left(\underset{i\in \{x\}}{\sum \;}\text{exp}\left(-G_{i}/RT\right)\right)$$

where the summation runs over all the conformers of the *x* species. The influence of the solvent on some of the studied species was introduced by means of an implicit solvent model, namely SMD.^60^ Indeed, accurate Gibbs free energies of solvation can be predicted using such models, provided that specific parameters are used to build At cavities. Hence, we have introduced an astatine Coulomb radius consistent with the 2018 revised Coulomb radii^61^ used in the SMD model for all other halogens. The Coulomb radii of F, Cl, Br and I were compared against different sets of radii: computed atomic radii,^62^ consistent van der Waals radii,^63^ revisited empirical covalent radii,^64^ and single-bond covalent radii^65^ (see Fig. S7 in ESI†). A strong linear relationship was obtained with the computed atomic radii by Rahm *et al*.^62^ (the associated coefficient of determination is 0.985 and the standard deviation on Coulomb radii is 0.07 Å). Using the computed atomic radius for At, we have extrapolated a value of 2.93 Å that we recommend as the Coulomb radius for building At cavities. The procedure used to compute free energies of aqueous solvation (*T* = 298.15 K, 1 mol L^−1^ standard state) was to perform, on top of previously optimized geometries, gas-phase and SMD single-point energy calculations using the TZVPD basis set.

For predicting the reactivity and selectivity of some studied species, several local descriptors have been determined from the total electron densities computed at the 2c-DFT/TZVPD level of theory. These computations were performed using the resolution of identity technique as implemented in the TURBOMOLE program.^66^ In the framework of conceptual density functional theory,^67^ maximal interactions with a nucleophile (respectively an electrophile) is expected when the quantity:

$$\text{Δ}f(r)=f{\left(r\right)}^{+}-f{\left(r\right)}^{-}$$

is maximal (respectively minimal). Δ *f* is the so-called dual descriptor, and *f* ^+^ and *f* ^−^ are, respectively, the electrophilic and nucleophilic Fukui functions:

$$\begin{array}{c} f{\left(r\right)}^{+}=\rho_{N+1}(r)-\rho_{N}(r)\\ f{\left(r\right)}^{-}=\rho_{N}(r)-\rho_{N-1}(r) \end{array}$$

where *ρ*_*N*_, *ρ*_*N*+1_ and *ρ*_*N*−1_ represent the electron density of the neutral species (*N* electrons) and of the corresponding anion (*N* + 1 electrons) and cation (*N* − 1 electrons) at the geometry of the neutral species. The related condensed-to-atom variants are written for the atomic site *k* of the studied species by exchanging the electron densities by the respective partial atomic charge *q*_*k*_:

$$\begin{array}{c} f_{k}^{+}=q_{k}(N)-q_{k}(N+1)\\ f_{k}^{-}=q_{k}(N-1)-q_{k}(N) \end{array}$$

The *q*_*k*_ atomic charges have been computed within the NPA model.^39^ Species reactivity and selectivity can therefore be analysed on the basis of atomic sites thanks to the atomic Δ*f*_*k*_ values of the dual descriptor. In order to compare the values of the dual descriptor for a given PhAt site in different XB complexes, however, it is necessary to normalize these values.^68^ The Fukui functions are evaluated on complexes with different numbers of electrons, but all include the PhAt fragment. We therefore propose to renormalize the condensed-to-atom Fukui indices as follows:

$$\begin{array}{c} \underset{k}{\sum \;}f_{k}^{+}=1\\ \underset{k}{\sum \;}f_{k}^{-}=1 \end{array}$$

where for each sum, the index *k* runs over all atoms of PhAt in the XB complex.

Note that the trends obtained with the PW6B95 and B3LYP methods were generally the same, both in the evolution of geometric, energy and reactivity descriptors. Hence, only the results obtained at the PW6B95/TZVPD level of theory are discussed throughout the text, unless stated otherwise.

## Supplementary Material

Supplementary Material

## Figures and Tables

**Table 1:** Relative stabilities (Δ*G*_r_), Boltzmann populations (*p*_i_) and Gibbs energy of solvation in the aqueous phase (Δ*G*_sol_) for the main complexes between OABG and the deprotonated selenocysteine fragment

Complexes	1	2	3	4	5	6	7
Δ*G*_r_ (kJ mol^−1^)	15.2	21.3	25.0	0	0.5	1.3	5.7
*p*_i_ (%)	0	0	0	40	32	24	4
Δ*G*_sol_ (kJ mol ^−1^)	−86.0	−86.8	−91.7	−86.0	−81.7	−99.2	−86.7
*p*_i_ (%)	0	0	0	1	0	99	0
