# Supplementary material for: In vivo stability of 211At-radiopharmaceuticals: on the impact of halogen bond formation
Source: RSC Med Chem. Author manuscript; Available in PMC 2025 Jan 30. (PMC10809332; doi:10.1039/d3md00579h)
Supplement: Supplementary Material [file EMS202158-supplement-Supplementary_Material.pdf]

Electronic Supplementary Material (ESI) for RSC Medicinal Chemistry.
This journal is © The Royal Society of Chemistry 2023

Electronic Supplementary Information (ESI)

In vivo stability of ^{211}At-radiopharmaceuticals: on the impact of halogen bond formation

Thibault Yssartier, Lu Liu, Sylvain Pardoue, Jean-Yves Le Questel, François Guérard, Gilles Montavon, Nicolas Galland

Table of contents:

Modelling of experimental data. 2

Table S1 Calculated interaction distances and angles of XBs between astatobenzene and nucleophilic sites of amino acids, at the PW6B95/AVDZ level of theory, while interaction energies are determined at the PW6B95/TZVPD level of theory 3

Fig. S1 Calculated electrostatic potential at the molecular surfaces, defined by 0.001 a.u. isovalue of the electron density, of iodobenzene and astatobenzene 4

Fig. S2 Calculated structures at the PW6B95/AVDZ level of theory for the most stable XB complexes between PhAt and nucleophilic sites of the asparagine, serine, cysteine, cystine, selenocysteine, phenylalanine, tryptophan and histidine #2 amino acids 4

Fig. S3 Astatine distribution ratios *D* as a function of the initial TMTU concentration in the organic phase 5

Fig. S4 Calculated interaction energy and charge transfer at the PW6B95/TZVPD level of theory, in the most stable XB complexes between PhAt and amino acid fragments 5

Fig. S5 NPA charges (in e) of the ring carbon atoms in PhAt and EAB calculated at the PW6B95/TZVPD level of theory 6

Fig. S6 Condensed-to-atom dual descriptor of C_{ipso} (Δf_C) and interaction energy calculated at the PW6B95/TZVPD level of theory for the most stable XB complexes between PhAt and amino acid fragments 6

Fig. S7 Correlation for F, Cl, Br and I between their Coulomb radii used in SMD model and four sets of radii 7

Supplementary structures. Cartesian coordinates (in angstroms) calculated at the PW6B95/AVDZ level of theory for all the complexes shown in the various figures, the Boltzmann populations come from the PW6B95/TZVPD calculations 8

Modelling of experimental data. The objective is to reproduce the experimental curves displaying D variations by a thermodynamic model considering the chemical equilibria occurring in the biphasic system.

In the model 1 of D supposing the formation of a XB complex in the aqueous phase, the following equilibria was considered:

$$\mathrm{EAB} \overset{D_1}{\Leftrightarrow} \overline{\mathrm{EAB}}$$

$$\mathrm{TMTU} \overset{D_2}{\Leftrightarrow} \overline{\mathrm{TMTU}}$$

$$\mathrm{EAB} + \mathrm{TMTU} \overset{K_\mathrm{a}}{\Leftrightarrow} \mathrm{EAB} \cdots \mathrm{TMTU}$$

The overlined notation means that these species belong to the organic phase. Finally, D can be expressed as:

$$D_{\mathrm{Model\ 1}} = \frac{D_1}{1 + K_\mathrm{a} \times [\mathrm{TMTU}] \times \frac{1}{2 + D_2}}$$

In the model 2 of D supposing the formation of a XB complex in the organic phase, the following equilibria was considered:

$$\mathrm{EAB} \overset{D_1}{\Leftrightarrow} \overline{\mathrm{EAB}}$$

$$\mathrm{TMTU} \overset{D_2}{\Leftrightarrow} \overline{\mathrm{TMTU}}$$

$$\overline{\mathrm{EAB}} + \overline{\mathrm{TMTU}} \overset{K_\mathrm{o}}{\Leftrightarrow} \overline{\mathrm{EAB} \cdots \mathrm{TMTU}}$$

Therefore, D can be expressed as:

$$D_{\mathrm{Model\ 2}} = D_1 \times (1 + K_\mathrm{o} \times [\mathrm{TMTU}] \times \frac{D_2}{2 + D_2})$$

Origin 9.0 was used to fit the experimental data, *i.e.* obtain the lines displayed in Fig. S2, in order to determine the unknown parameters:

Model 1	Model 2
$D_1 = 10^{2.35}$	$D_1 = 10^{2.35}$
$D_2 = 10^{-0.37}$ [a]	$D_2 = 10^{-0.37}$ [a]
$K_\mathrm{a} = 10^{0.5}$	$K_\mathrm{o} = 10^{1.0}$

[a] The D_2 value was determined experimentally in a previous study.[20]

Table S1 Calculated interaction distances and angles of XBs between astatobenzene and nucleophilic sites of amino acids, at the PW6B95/AVDZ level of theory, while interaction energies are determined at the PW6B95/TZVPD level of theory. [a]

sites		E_{int} (kJ/mol)	d_{int} (Å)	θ (°)
π	phenylalanine	–6.4	-	167.5
	tyrosine	–7.1	-	162.5
	tryptophan	–8.3	-	162.1
	histidine	–8.7	-	160.2
N (sp^2)	peptide backbone	–5.4	3.368	174.4
	asparagine	–6.6	3.315	176.7
	histidine	–20.5	2.980	179.6
O (sp^2)	peptide backbone	–16.6	2.972	178.9
	asparagine	–18.5	2.957	176.7
O (sp^3)	tyrosine	–9.5	3.160	179.0
	serine	–12.6	3.049	178.0
	aspartate	–77.9	2.597	175.0
S (sp^3)	cystine	–9.9	3.505	172.2
	cysteine	–11.7	3.496	175.6
	methionine	–16.7	3.405	171.9
Se (sp^3)	selenocysteine	–12.1	3.585	175.3
	deprotonated selenocysteine	–86.6	3.075	177.5

[a] For a given type of interaction, there may be N stable conformers. In this case, the value of a property, A, is calculated from a weighting of each conformer i by its Boltzmann population p_i: $A = \sum_i^N \left(\frac{p_i}{\sum_j^N p_j} A_i\right)$.

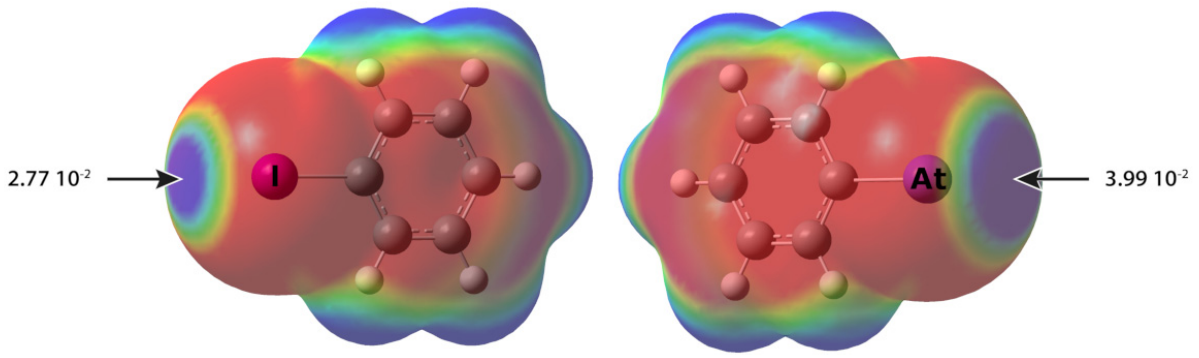

Fig. S1 Calculated electrostatic potential at the molecular surfaces, defined by 0.001 a.u. isovalue of the electron density, of iodobenzene (left) and astatobenzene (right). Values of $V_{S,max}$ in a.u. come from PW6B95/TZVPD calculations. Color code: from red (lowest values) to blue (highest values).

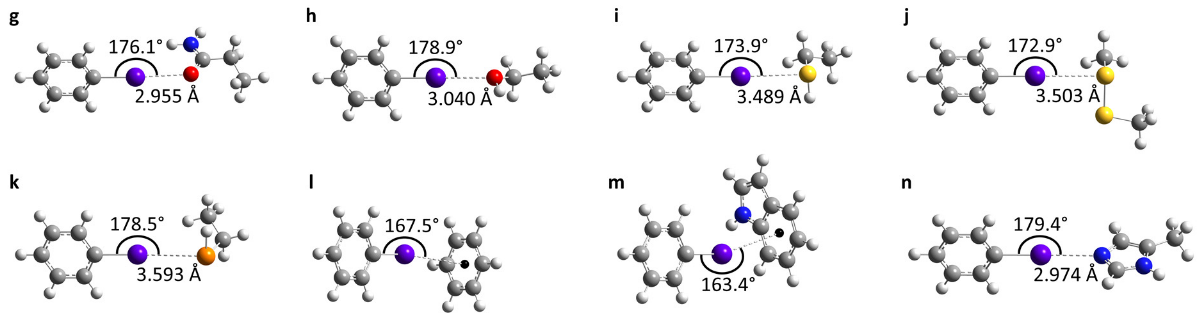

Fig. S2 Calculated structures at the PW6B95/AVDZ level of theory for the most stable XB complexes between PhAt and nucleophilic sites of the asparagine (g), serine (h), cysteine (i), cystine (j), selenocysteine (k), phenylalanine (l), tryptophan (m) and histidine #2 (n) amino acids. Atom color codes: purple for At, red for O, blue for N, yellow for S, orange for Se, gray for C, and white for H.

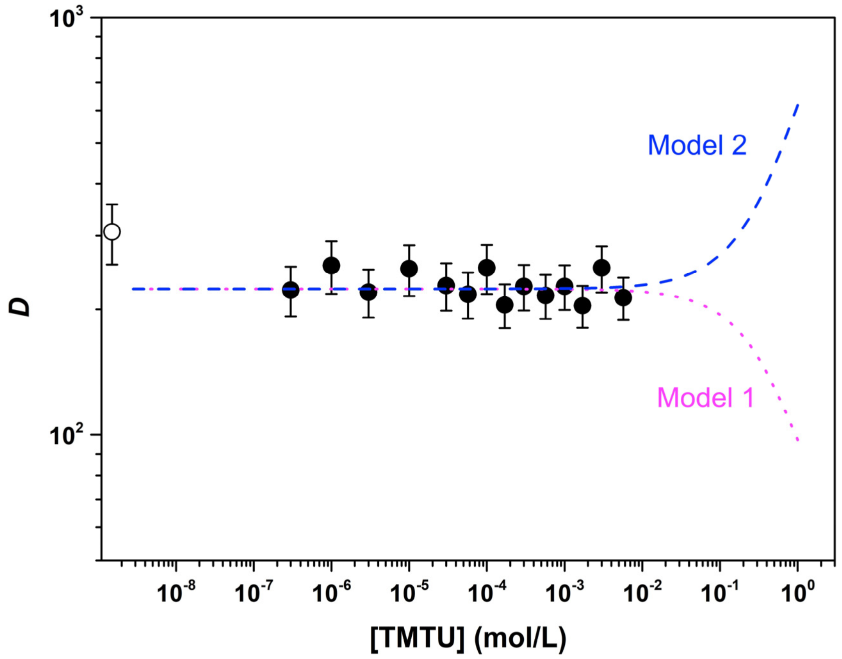

Fig. S3 Astatine distribution ratios D as a function of the initial TMTU concentration in the organic phase. The hollows symbol indicates the D value in absence of TMTU in the biphasic system. The pink dotted line is the model 1 of D considering the formation of a XB complex in the aqueous phase with $K_{EAB} = 10^{0.5}$, while the blue dashed line is the model 2 of D considering the formation of a XB complex in the organic phase with $K_{EAB} = 10^{1.0}$.

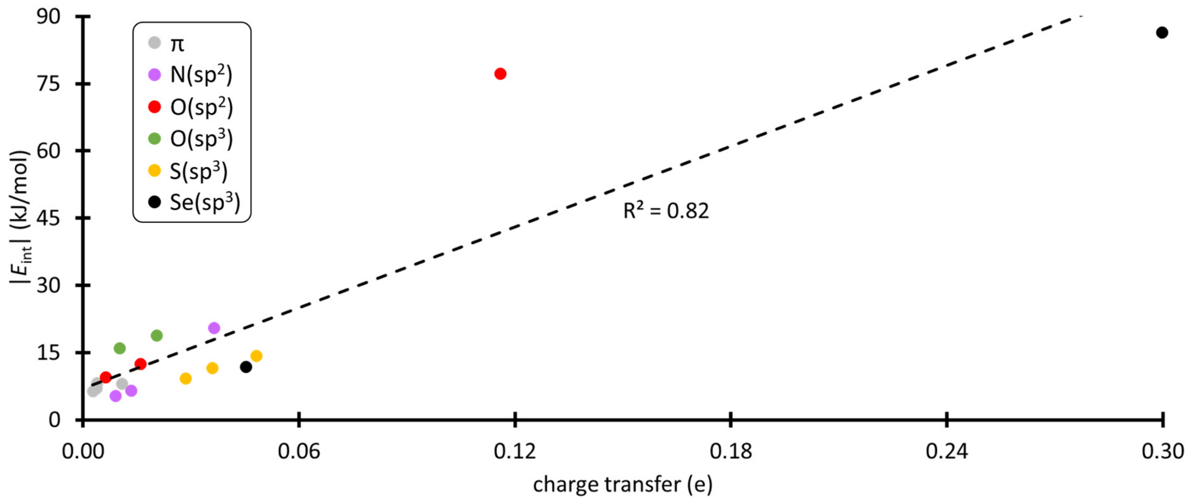

Fig. S4 Calculated interaction energy and charge transfer at the PW6B95/TZVPD level of theory, in the most stable XB complexes between PhAt and amino acid fragments (data sorted according to the type of acceptor site).

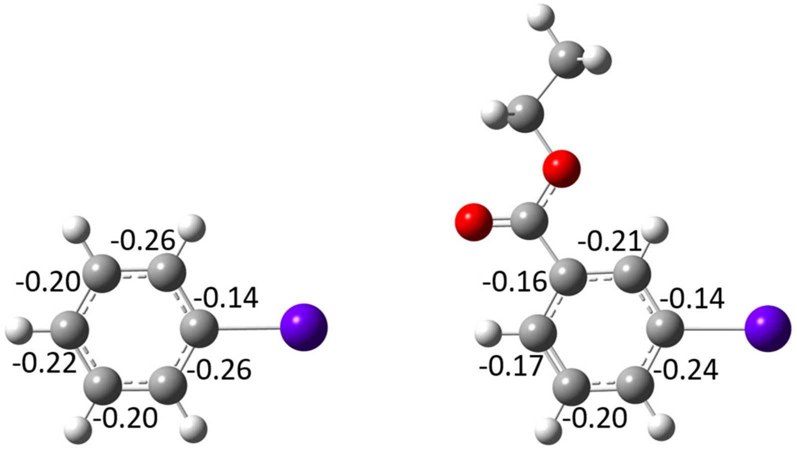

Fig. S5 NPA charges (in e) of the ring carbon atoms in PhAt and EAB calculated at the PW6B95/TZVPD level of theory. EAB has four stable conformers and the presented values are calculated from a weighting of each conformer *i* by its Boltzmann population p_i: $q = \sum_i^4 p_i \, q_i$.

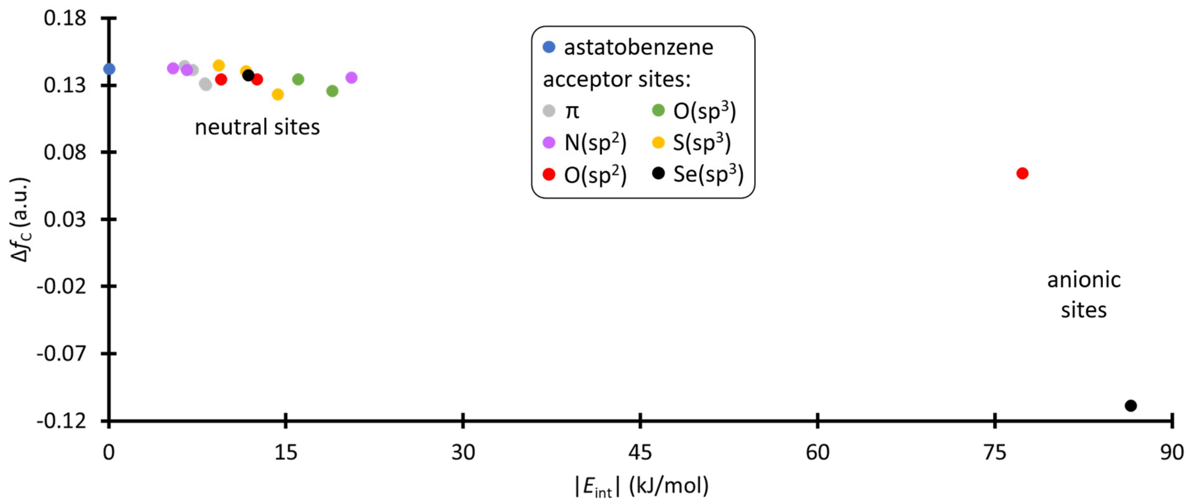

Fig. S6 Condensed-to-atom dual descriptor of C_{ipso} (Δf_C) and interaction energy calculated at the PW6B95/TZVPD level of theory for the most stable XB complexes between PhAt and amino acid fragments (data sorted according to the type of acceptor site).

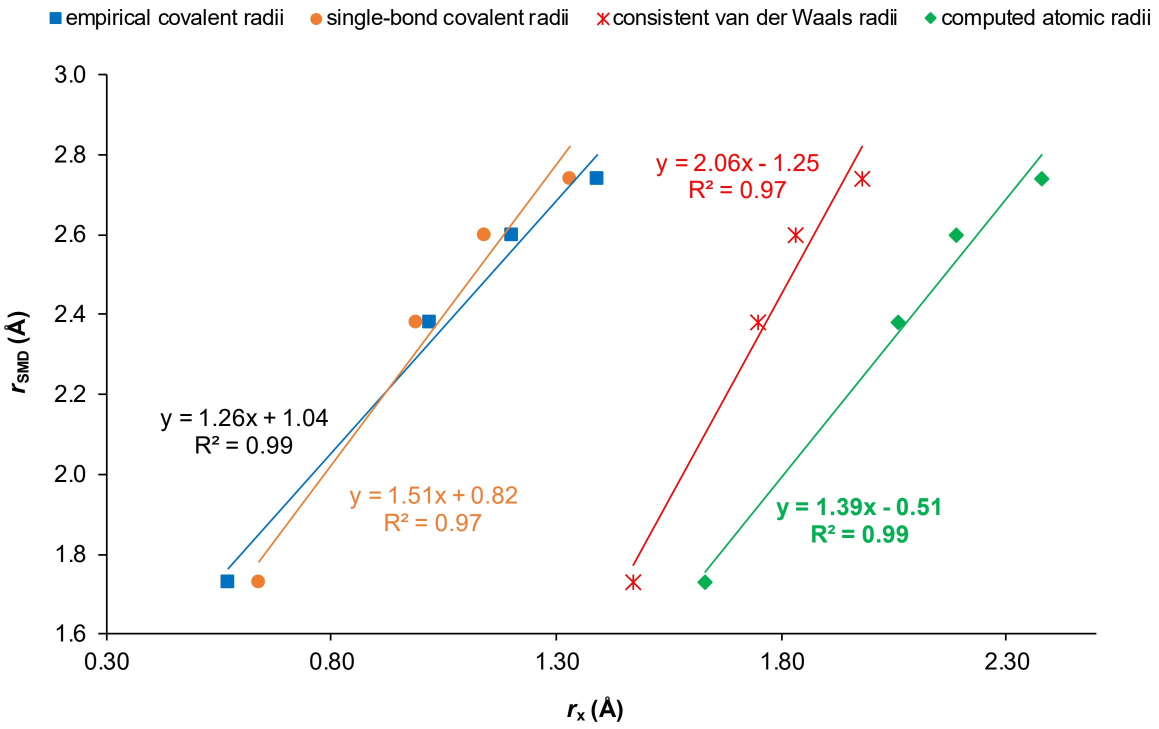

Fig. S7 Correlation for F, Cl, Br and I between their Coulomb radii used in SMD model and four sets of radii (computed atomic radii, consistent van der Waals radii, revisited empirical covalent radii and single-bond covalent radii).

Electronic Supplementary Information (ESI)

Supplementary structures. Cartesian coordinates (in angstroms) calculated at the PW6B95/AVDZ level of theory for all the complexes shown in the various figures, the Boltzmann populations come from the PW6B95/TZVPD calculations.

Complex **a**, pop=24%

C	4.046616	0.175116	0.000000
O	2.883033	0.559633	-0.000002
N	4.388692	-1.135497	0.000002
H	5.367553	-1.364906	0.000004
C	3.421614	-2.209657	0.000003
H	3.532176	-2.835576	0.891500
H	3.532178	-2.835578	-0.891494
H	2.425412	-1.765508	0.000001
C	5.192014	1.153297	0.000000
H	6.173146	0.670709	0.000002
H	5.104110	1.793251	0.881592
H	5.104112	1.793249	-0.881594
C	-2.310012	-0.026448	0.000000
C	-2.999365	-0.114045	1.205920
C	-4.381065	-0.290454	1.202181
C	-5.075235	-0.379265	0.000002
C	-4.381066	-0.290451	-1.202178
C	-2.999367	-0.114041	-1.205919
H	-2.465712	-0.045009	2.150025
H	-4.914738	-0.357875	2.147347
H	-6.153276	-0.516550	0.000002
H	-4.914741	-0.357869	-2.147344
H	-2.465715	-0.045003	-2.150024
At	-0.084640	0.257809	-0.000001

Complex **b**, pop=80%

C	5.991298	0.002633	-0.074342
C	4.900864	-0.859112	-0.132505
C	3.613318	-0.343766	-0.018925
C	3.409912	1.022275	0.151605
C	4.508414	1.870331	0.207949
C	5.802970	1.369522	0.095840
H	6.995562	-0.403439	-0.163469
H	5.050564	-1.929276	-0.265777
H	2.395870	1.402233	0.237618
H	4.348307	2.937087	0.341224
H	6.656597	2.039225	0.140780
O	2.503838	-1.142729	-0.068336
H	2.768525	-2.057597	-0.190252
At	-0.558948	-0.365163	-0.018042
C	-2.740004	0.149770	0.002391
C	-3.372535	0.513983	-1.181403
C	-3.449001	0.103293	1.197930
C	-4.728055	0.834156	-1.164363
H	-2.818243	0.550343	-2.115019
C	-4.804253	0.425182	1.204492
H	-2.954460	-0.181052	2.122473
C	-5.446261	0.790768	0.026012
H	-5.221613	1.119015	-2.090426
H	-5.357668	0.388647	2.139767
H	-6.503570	1.041265	0.035270

Complex **c**, pop=49%

C	3.328824	-0.604560	-0.000018
O	2.940471	-1.783242	-0.000050
O	2.605292	0.442222	0.000010
C	4.848300	-0.366764	-0.000009
H	5.250394	-0.895855	0.873187
H	5.250395	-0.895800	-0.873236
C	5.278261	1.089679	0.000037
H	6.372828	1.183971	0.000041
H	4.882912	1.609559	-0.878085
H	4.882910	1.609505	0.878190
At	0.023936	0.109480	0.000001
C	-2.274680	0.018753	0.000001
C	-2.943251	-1.208487	0.000029
C	-3.048763	1.182153	-0.000027
C	-4.335430	-1.273812	0.000029
H	-2.367281	-2.132272	0.000052
C	-4.441451	1.125072	-0.000028
H	-2.556542	2.153318	-0.000050
C	-5.092703	-0.105449	0.000001
H	-4.830810	-2.243719	0.000052
H	-5.020457	2.047591	-0.000050
H	-6.179660	-0.153468	0.000001

Complex **d**, pop=74%

C	3.691085	-0.954259	1.351430
H	3.617804	-2.028385	1.163881
H	4.643195	-0.740668	1.846190
H	2.854841	-0.644605	1.986264
S	3.609174	-0.128171	-0.258355
C	3.715826	1.594729	0.289669
H	4.668091	1.780159	0.795328
H	3.657682	2.219352	-0.605178
H	2.879898	1.840567	0.952287
At	0.203603	-0.101643	-0.254497
C	-2.024353	0.026529	0.045513
C	-2.628373	1.265959	0.228880
C	-2.788996	-1.135365	0.046157
C	-4.007120	1.339801	0.414227
H	-2.033273	2.175205	0.227927
C	-4.167157	-1.052898	0.232152
H	-2.319632	-2.104813	-0.097663
C	-4.778923	0.182624	0.416455
H	-4.476811	2.310125	0.557007
H	-4.762579	-1.962829	0.231888
H	-5.854195	0.243567	0.561070

Complex **e**, pop=63%

C	-4.987148	-0.312291	0.000001
C	-3.700318	-0.782972	0.000002
C	-3.565046	1.364282	-0.000003
N	-4.883013	1.058332	-0.000002
H	-5.644931	1.714807	-0.000004
H	-5.941126	-0.817435	0.000002
H	-3.198444	2.380763	-0.000005
N	-2.826874	0.280008	-0.000000
C	-3.218443	-2.190093	0.000005
H	-2.600866	-2.390342	0.881626
H	-4.059415	-2.887912	0.000006
H	-2.600866	-2.390346	-0.881615
At	0.150944	0.112419	-0.000000
C	2.399912	-0.000401	0.000000
C	3.037110	-1.238346	-0.000004
C	3.157857	1.167511	0.000004
C	4.428497	-1.305715	-0.000004
H	2.453651	-2.155500	-0.000007
C	4.548994	1.095368	0.000005
H	2.669066	2.138445	0.000008
C	5.187997	-0.140258	0.000001
H	4.918378	-2.276735	-0.000007
H	5.133594	2.012490	0.000008
H	6.273448	-0.194757	0.000001

Complex **f**, pop=28%

C	4.770837	-1.671412	-0.000132
C	3.287285	-1.349375	-0.000095
H	2.805372	-1.783763	0.883436
H	2.805355	-1.783643	-0.883675
H	5.260082	-1.245197	0.883391
H	4.947580	-2.758733	-0.000206
H	5.260063	-1.245079	-0.883608
At	-0.112313	0.242713	0.000018
C	-2.438784	-0.114438	-0.000008
C	-3.337304	0.955442	-0.000122
C	-2.970860	-1.406221	0.000093
C	-4.716143	0.747402	-0.000135
H	-2.957049	1.976752	-0.000203
C	-4.347838	-1.625860	0.000080
H	-2.298697	-2.264138	0.000184
C	-5.228677	-0.547328	-0.000034
H	-5.393820	1.600629	-0.000225
H	-4.735066	-2.644329	0.000161
H	-6.303941	-0.714182	-0.000043
Se	2.942519	0.597111	0.000040

Complex **g**, pop=54%

C	5.597027	-0.835920	-0.381849
H	5.120832	-1.033302	-1.344754
H	5.376264	-1.681340	0.276007
H	6.679816	-0.784084	-0.526011
C	5.078171	0.461627	0.208861
H	5.528929	0.663475	1.188050
H	5.348435	1.311089	-0.432453
C	3.570960	0.480649	0.341087
O	2.848451	-0.268523	-0.304862
N	3.068441	1.395508	1.203942
H	3.663116	2.008711	1.732139
H	2.068062	1.486609	1.293455
At	-0.102456	-0.180249	-0.176160
C	-2.334047	0.005223	0.017050
C	-3.030216	0.893816	-0.796879
C	-3.017054	-0.769790	0.949561
C	-4.413362	1.005664	-0.674859
H	-2.501555	1.499846	-1.527820
C	-4.400259	-0.652942	1.066169
H	-2.478102	-1.466296	1.586297
C	-5.101362	0.233973	0.255767
H	-4.952911	1.700896	-1.313732
H	-4.929509	-1.261339	1.795801
H	-6.180485	0.322741	0.348462

Complex **h**, pop=52%

C	-5.547057	0.140857	0.542371
C	-4.076267	0.491876	0.571322
H	-3.632680	0.223263	1.534675
H	-3.930777	1.572410	0.431818
H	-5.691976	-0.931807	0.702328
H	-6.082523	0.686323	1.326985
H	-5.999312	0.411523	-0.419399
H	-3.672715	-0.039035	-1.269912
O	-3.322691	-0.233689	-0.396959
At	-0.295527	-0.100722	-0.155980
C	1.935003	0.031394	0.048998
C	2.567543	1.267605	-0.035696
C	2.678940	-1.125086	0.260319
C	3.952573	1.343482	0.092187
H	1.988848	2.172307	-0.200768
C	4.063544	-1.040494	0.387093
H	2.187388	-2.091816	0.326784
C	4.703326	0.191765	0.303575
H	4.443830	2.311334	0.025444
H	4.641960	-1.946406	0.552171
H	5.783647	0.254367	0.402845

Complex **i**, pop=45%

C	5.136886	-1.472083	0.687005
C	3.780794	-0.806831	0.801411
S	3.560006	0.619595	-0.325028
H	3.633558	-0.378313	1.797871
H	2.963882	-1.513790	0.629903
H	5.943469	-0.756754	0.871635
H	5.219826	-2.285477	1.417201
H	5.283403	-1.899173	-0.310514
H	3.674387	-0.070493	-1.480526
At	0.088745	0.304565	-0.166068
C	-2.111959	-0.107167	0.044857
C	-2.686764	-1.168313	-0.646435
C	-2.888912	0.702266	0.866790
C	-4.050471	-1.418739	-0.511566
H	-2.080987	-1.800976	-1.289382
C	-4.251832	0.444535	0.995682
H	-2.441256	1.531992	1.406911
C	-4.835273	-0.614645	0.308350
H	-4.498094	-2.248700	-1.053110
H	-4.857641	1.078688	1.638555
H	-5.898764	-0.812706	0.411098

Complex **j**, pop=67%

C	-5.288952	1.338199	-0.189315
H	-5.741129	0.774666	-1.008107
H	-5.672788	0.993891	0.774046
H	-5.517337	2.402624	-0.307246
S	-3.475264	1.210802	-0.254481
S	-3.178726	-0.832886	-0.109141
C	-3.154547	-1.098396	1.691445
H	-2.944479	-2.163260	1.836054
H	-2.361359	-0.502576	2.148718
H	-4.125288	-0.857876	2.131087
At	0.282759	-0.300708	-0.182349
C	2.475710	0.140129	0.028741
C	3.410245	-0.841312	-0.283429
C	2.887643	1.393062	0.469712
C	4.768724	-0.562675	-0.151405
H	3.088851	-1.820020	-0.628791
C	4.248278	1.662472	0.598631
H	2.157644	2.160211	0.712726
C	5.190677	0.687339	0.289175
H	5.498345	-1.330901	-0.396138
H	4.568989	2.642690	0.943187
H	6.251221	0.901201	0.390721

Complex **k**, pop=43%

C	3.211415	2.236607	0.555995
C	3.966365	0.957111	0.842636
H	3.831597	0.634143	1.879790
H	5.038993	1.063168	0.663318
H	2.139683	2.116174	0.739475
H	3.583542	3.043733	1.198774
H	3.343359	2.549276	-0.485013
H	3.585033	0.042881	-1.509894
At	-0.219734	-0.187386	-0.094730
C	-2.456421	0.038778	0.023749
C	-3.127591	0.782336	-0.941210
C	-3.158290	-0.567037	1.060495
C	-4.511819	0.919168	-0.864755
H	-2.580800	1.255706	-1.752164
C	-4.542407	-0.425013	1.129408
H	-2.635617	-1.149116	1.814610
C	-5.221682	0.317041	0.168816
H	-5.034613	1.500763	-1.620356
H	-5.089233	-0.899712	1.940666
H	-6.301439	0.425490	0.225361
Se	3.348220	-0.594372	-0.202745

Complex **l**, pop=65%

C	3.642161	-0.179565	1.218971
C	3.619569	1.058996	0.581725
C	3.652800	1.126783	-0.809066
C	3.709225	-0.042851	-1.561782
C	3.732084	-1.280346	-0.924792
C	3.698464	-1.349070	0.465098
H	3.613540	-0.232860	2.304259
H	3.574188	1.972076	1.169793
H	3.630685	2.092956	-1.306420
H	3.729931	0.010229	-2.647142
H	3.771106	-2.193474	-1.512986
H	3.711701	-2.315449	0.962323
At	-0.032496	0.116165	0.161325
C	-2.259683	-0.032298	-0.033304
C	-2.870764	-1.281015	-0.014712
C	-3.015933	1.126205	-0.171085
C	-4.255868	-1.366539	-0.135762
H	-2.277962	-2.184852	0.093114
C	-4.400270	1.029214	-0.291433
H	-2.536375	2.100760	-0.185401
C	-5.022803	-0.214479	-0.274129
H	-4.733876	-2.343062	-0.121149
H	-4.991718	1.935234	-0.399190
H	-6.102963	-0.285763	-0.368203

Complex **m**, pop=50%

C	3.370873	0.724642	-0.671836
C	3.257829	0.290293	0.671138
C	3.040054	-1.046450	1.008630
C	2.933160	-1.956612	-0.031501
C	3.042661	-1.547578	-1.373778
C	3.261340	-0.221426	-1.701003
C	3.575880	2.140508	-0.635745
C	3.578990	2.508010	0.680691
H	2.954204	-1.365411	2.044196
H	2.761658	-3.005613	0.194253
H	2.949349	-2.289192	-2.162234
H	3.339602	0.085383	-2.740698
H	3.703415	2.805450	-1.478935
H	3.701949	3.484729	1.128164
N	3.391428	1.398154	1.469350
H	3.334424	1.402507	2.471970
At	-0.459138	-0.465910	0.075436
C	-2.608230	0.162503	-0.026220
C	-2.950581	1.336812	-0.687720
C	-3.586834	-0.624361	0.571281
C	-4.287181	1.724207	-0.749669
H	-2.185095	1.950337	-1.154431
C	-4.920600	-0.228262	0.503719
H	-3.318550	-1.541936	1.087198
C	-5.273966	0.944569	-0.155352
H	-4.553888	2.642393	-1.267512
H	-5.685165	-0.844427	0.970805
H	-6.315571	1.249941	-0.206111

Complex **n**, pop=96%

C	-3.571146	-0.038519	-0.843373
C	-4.853987	-0.023158	-0.362506
C	-3.375128	0.062708	1.282074
N	-2.662896	0.015034	0.183967
H	-3.254862	-0.085442	-1.876604
H	-2.987467	0.112246	2.289542
N	-4.704162	0.042075	1.003982
C	-6.175425	-0.062635	-1.038614
H	-6.761857	-0.938902	-0.739219
H	-6.029258	-0.112648	-2.119525
H	-6.772162	0.830992	-0.822484
H	-5.448901	0.069647	1.680331
At	0.307721	0.006883	0.039519
C	2.558000	-0.004046	-0.048231
C	3.260437	1.195552	-0.127730
C	3.251444	-1.211224	-0.023426
C	4.652397	1.185483	-0.182038
H	2.727247	2.142592	-0.147931
C	4.643390	-1.216395	-0.077953
H	2.711140	-2.152427	0.038176
C	5.347479	-0.019258	-0.157322
H	5.193545	2.126885	-0.244173
H	5.177440	-2.163673	-0.058277
H	6.433476	-0.025157	-0.199907

Complex **1**, pop=0%

C	3.429285	-1.364679	-0.187408
C	4.783007	-1.038630	-0.130561
C	5.175647	0.239503	0.249141
C	4.206910	1.182323	0.572832
C	2.847907	0.862504	0.520503
C	2.451079	-0.428164	0.134893
H	3.136951	-2.368972	-0.485309
H	5.530280	-1.787747	-0.382025
H	6.228930	0.501648	0.297869
H	4.508921	2.182535	0.881413
C	1.844890	1.926453	0.898917
H	1.113739	1.505302	1.594952
H	2.351444	2.760349	1.393813
N	1.109052	2.431799	-0.267835
H	1.646147	2.483840	-1.120542
C	-0.216670	2.328221	-0.431629
N	-0.731503	2.303762	-1.677341
N	-1.081451	2.317290	0.577523
H	-1.894310	1.669435	0.396751
H	-0.707997	2.317151	1.512648
H	-0.171350	1.850604	-2.383570
H	-1.715280	2.043057	-1.708986
At	0.180354	-0.907657	-0.038539
Se	-2.910694	-0.190778	-0.529062
C	-3.929079	-0.507284	1.139380
C	-5.177511	0.348594	1.226228
H	-4.187938	-1.570038	1.150496
H	-3.268803	-0.322203	1.993362
H	-5.838361	0.161906	0.373684
H	-5.736740	0.132145	2.146971
H	-4.927571	1.415321	1.222228

Complex **2**, pop=0%

C	3.316164	-1.422854	-0.187303
C	4.675205	-1.126651	-0.099255
C	5.087077	0.142555	0.290095
C	4.132053	1.106428	0.592073
C	2.768077	0.815649	0.508395
C	2.350826	-0.465864	0.113198
H	3.009475	-2.420804	-0.492433
H	5.411691	-1.891828	-0.333867
H	6.144494	0.381494	0.363066
H	4.448856	2.099888	0.907733
C	1.779649	1.901247	0.863508
H	1.024201	1.497635	1.544019
H	2.292274	2.726370	1.366675
N	1.080273	2.417935	-0.321328
H	1.635609	2.448086	-1.163387
C	-0.243151	2.344736	-0.511708
N	-0.732392	2.323692	-1.768584
N	-1.128329	2.366572	0.479231
H	-0.779786	2.355990	1.424234
H	-1.958389	1.751959	0.291792
H	-0.171436	1.843870	-2.456429
H	-1.721367	2.092505	-1.820420
At	0.063638	-0.886393	-0.107586
H	-3.674766	0.000484	3.284169
C	-3.213293	0.273078	2.325392
C	-3.848000	-0.486453	1.176638
H	-3.337979	1.355641	2.197395
H	-2.140295	0.052336	2.394936
H	-4.904473	-0.219498	1.076808
H	-3.795467	-1.564328	1.354729
Se	-3.007541	-0.159266	-0.586636

Complex **3**, pop=0%

C	-3.265011	-1.332038	0.712763
C	-4.596014	-0.997265	0.954998
C	-5.067883	0.266723	0.620339
C	-4.200989	1.186650	0.042291
C	-2.865983	0.856890	-0.205163
C	-2.387561	-0.419497	0.133183
H	-2.911520	-2.326000	0.977762
H	-5.264355	-1.728596	1.403808
H	-6.104687	0.535161	0.803380
H	-4.567274	2.175589	-0.231271
C	-1.976092	1.898466	-0.841125
H	-1.416780	1.449422	-1.667025
H	-2.580804	2.719116	-1.237998
N	-0.999504	2.437465	0.116569
H	-1.335725	2.513763	1.065112
C	0.328187	2.322339	-0.007776
N	1.098219	2.314367	1.098682
N	0.952695	2.295047	-1.180785
H	1.792803	1.672066	-1.180793
H	0.390483	2.289880	-2.015941
H	0.702434	1.864240	1.910455
H	2.064139	2.050258	0.923751
At	-0.132874	-0.912955	-0.222053
Se	2.970136	-0.308286	-0.562445
C	3.315365	-0.340757	1.388447
C	4.403769	0.626264	1.815323
H	2.374644	-0.139300	1.919076
H	3.603607	-1.365791	1.639254
H	4.143257	1.667423	1.582623
H	4.593854	0.559393	2.895432
H	5.338979	0.411470	1.289076

Complex **4**, pop=40%

C	1.690552	2.366364	-0.479303
C	0.698905	3.339216	-0.505579
C	-0.566290	3.055243	0.000013
C	-0.832958	1.801459	0.526793
C	0.150339	0.804165	0.565308
C	1.408674	1.112049	0.054860
H	2.677729	2.586821	-0.874273
H	0.920244	4.317901	-0.923111
H	-1.346520	3.810834	-0.015372
H	-1.822471	1.570974	0.922039
C	-0.234727	-0.530486	1.165343
H	0.597822	-1.239561	1.117239
H	-0.469180	-0.398317	2.225314
N	-1.414934	-1.108034	0.559740
H	-2.384047	-0.851357	0.927077
C	-1.438245	-1.670665	-0.635754
N	-2.603843	-2.152424	-1.083788
N	-0.321949	-1.800030	-1.374810
H	-0.338168	-2.326041	-2.229633
H	0.557712	-1.408077	-1.078478
H	-3.443068	-1.832008	-0.542136
H	-2.709843	-2.314865	-2.071572
At	3.112918	-0.370974	0.046459
Se	-4.477109	-0.331964	0.870813
C	-4.147535	0.900774	-0.646413
C	-5.167047	0.748831	-1.759933
H	-4.163241	1.925454	-0.258382
H	-3.135000	0.724284	-1.038466
H	-6.179439	0.929724	-1.385652
H	-4.972034	1.453517	-2.581250
H	-5.151776	-0.267989	-2.169980

Complex **5**, pop=32%

C	-2.164955	2.363524	0.218273
C	-1.343816	3.481442	0.296030
C	0.021136	3.358741	0.052095
C	0.556116	2.121392	-0.269610
C	-0.252912	0.980951	-0.361902
C	-1.614271	1.127207	-0.108907
H	-3.228941	2.456710	0.414362
H	-1.775100	4.445924	0.550862
H	0.670325	4.227636	0.111536
H	1.625735	2.018345	-0.455249
C	0.417229	-0.325149	-0.733267
H	-0.292257	-1.157594	-0.692893
H	0.773181	-0.272481	-1.766001
N	1.576289	-0.614629	0.080410
H	2.528532	-0.233232	-0.205718
C	1.512871	-0.962162	1.355244
N	2.657770	-1.078511	2.032402
N	0.334360	-1.248918	1.942333
H	0.303565	-1.454625	2.924840
H	-0.537710	-1.019560	1.491964
H	3.490533	-0.630921	1.569140
H	2.630622	-1.142156	3.036328
At	-3.054045	-0.611090	-0.188464
Se	4.538169	0.582107	-0.067629
C	5.417613	-0.939215	-0.982895
C	4.496343	-2.111913	-1.259976
H	5.833064	-0.553879	-1.919159
H	6.258771	-1.254986	-0.357968
H	3.669639	-1.814283	-1.915922
H	5.034768	-2.934529	-1.751369
H	4.060295	-2.503914	-0.332514

Complex **6**, pop=24%

C	4.149474	0.838668	-0.398755
C	4.718669	2.107406	-0.467246
C	3.962604	3.227547	-0.145342
C	2.637669	3.072165	0.242512
C	2.047771	1.808792	0.319802
C	2.823997	0.693389	-0.007669
H	4.745461	-0.034996	-0.645845
H	5.756873	2.212555	-0.770685
H	4.401184	4.219965	-0.194736
H	2.041173	3.945745	0.497539
C	0.605987	1.693822	0.729405
H	0.496888	0.896190	1.474896
H	0.281816	2.637473	1.185209
N	-0.218652	1.367540	-0.422023
H	0.178675	1.502990	-1.336726
C	-1.451562	0.829864	-0.348181
N	-2.069520	0.689324	0.813467
H	-3.013149	0.190427	0.790248
H	-1.688716	1.082545	1.655549
At	1.993488	-1.392499	0.103380
N	-2.083872	0.442935	-1.454557
H	-6.198899	1.011620	-1.273635
C	-5.571086	1.061643	-0.378210
Se	-4.761474	-0.728627	-0.120392
C	-6.391478	1.512462	0.815595
H	-4.771646	1.786077	-0.584343
H	-5.770417	1.577313	1.716066
H	-6.845097	2.498551	0.638548
H	-7.194944	0.798704	1.024826
H	-3.005361	-0.055685	-1.301215
H	-1.558189	0.293154	-2.299360

Complex **7**, pop=4%

C	1.708605	2.370740	-0.420172
C	0.720821	3.345856	-0.480718
C	-0.571448	3.052734	-0.054807
C	-0.866449	1.788469	0.429509
C	0.112570	0.789628	0.504414
C	1.397017	1.105566	0.070463
H	2.715963	2.597344	-0.756359
H	0.965551	4.332782	-0.864319
H	-1.349963	3.808917	-0.100357
H	-1.877382	1.548347	0.758341
C	-0.304482	-0.555805	1.058069
H	0.524858	-1.269447	1.023951
H	-0.576004	-0.446644	2.111882
N	-1.465643	-1.107333	0.396517
H	-2.447115	-0.855241	0.739099
C	-1.450733	-1.637000	-0.813392
N	-2.602809	-2.100595	-1.314721
N	-0.313042	-1.739846	-1.525012
H	-0.293751	-2.287533	-2.366399
H	0.566009	-1.404933	-1.163811
H	-3.456028	-1.767413	-0.805314
H	-2.674584	-2.211294	-2.313272
At	3.092088	-0.386248	0.111919
Se	-4.572624	-0.488816	0.790072
C	-4.659593	1.187707	-0.263769
C	-3.986869	1.110819	-1.621922
H	-5.721004	1.424223	-0.386064
H	-4.221764	1.995196	0.334396
H	-4.439024	0.319841	-2.231753
H	-4.084252	2.058031	-2.171690
H	-2.915933	0.890784	-1.527438
